# The ethanolic extract of *Curcuma longa* grown in Korea exhibits anti-neuroinflammatory effects by activating of nuclear transcription factor erythroid-2-related factor 2/heme oxygenase-1 signaling pathway

**DOI:** 10.1186/s12906-022-03825-5

**Published:** 2022-12-30

**Authors:** Kwan-Woo Kim, Young-Seob Lee, Dahye Yoon, Geum-Soog Kim, Dae Young Lee

**Affiliations:** Department of Herbal Crop Research, National Institute of Horticultural and Herbal Sciences, Rural Development Administration, 27709 Eumseong, Republic of Korea

**Keywords:** *Curcuma longa*, Anti-neuroinflammation, BV2 microglial cells, Nuclear factor kappa B, Mitogen-activated protein kinases, Heme oxigenase-1

## Abstract

**Background:**

*Curcuma longa* has been used as spices, food preservative, coloring material, and traditional medicine. This plant also has long been used for a variety of diseases including dyslipidemia, stomach disorders, arthritis, and hepatic diseases. The aim of the present investigation was to examine the anti-neuroinflammatory effects of the 50% ethanolic extract of *C. longa* in lipopolysaccharide (LPS)-induced BV2 microglial cells.

**Methods:**

Griess reaction was employed to measure the production of nitric oxide (NO), and the levels of prostaglandin E2 (PGE_2_) and pro-inflammatory cytokines such as interleukin 1-beta (IL-1β), IL-6 and tumor necrosis factor-α (TNF-α) were determined by using profit ELISA kits. Western blotting was used to determine the expression of inducible nitric oxide synthase (iNOS), cyclooxygenase-2 (COX-2), nuclear factor kappa B (NF-κB), mitogen activated protein kinases (MAPKs), heme oxygenase-1 (HO-1) and nuclear factor erythroid-2-related factor 2 (Nrf2).

**Results:**

Pre-treatment with CLE inhibited the overproduction and overexpression of pro-inflammatory mediators including NO, PGE_2_, iNOS, COX-2, and pro-inflammatory cytokines such as IL-1β, IL-6 and TNF-α in LPS-induced BV2 cells. In addition, CLE suppressed the activation of the NF-κB and three MAPK signaling pathways. Treatment with CLE induced HO-1 protein expression by activating Nrf2 pathway, and inhibiting the HO-1 expression reversed the anti-inflammatory effect of CLE.

**Conclusion:**

CLE showed anti-neuroinflammatory effects against LPS-induced microglial cells activation through the inhibition of production and expression of pro-inflammatory mediators by negative regulation of the NF-κB and MAPK signaling pathways. These anti-neuroinflammatory effects of CLE were mediated by HO-1/Nrf2 signaling pathway. Taken together, the present study suggests a potent effect of CLE to prevent neuroinflammatory diseases. It is necessary to perform additional efficacy evaluation through in vivo experiments.

**Supplementary Information:**

The online version contains supplementary material available at 10.1186/s12906-022-03825-5.

## Background

Microglial cells are a type of macrophage present in the brain, which accounts for about 5–20% of glial cells and have primary immune defense functions in the central nervous system (CNS) [1]. The cells play a role in maintaining brain homeostasis, and when they detect minor pathological changes such as invasion of external pathogens or damage to nerve cells, they quickly activate and secrete inflammatory factors such as nitric oxide (NO), prostaglandin E2 (PGE_2_), and inflammatory cytokines including interleukins (ILs) and tumor necrosis factors (TNFs) [2]. However, prolonged activated microglial cells produce excessive amounts of inflammatory mediators, which cause collapse of homeostasis and oxidative damage to cell membrane, proteins, and DNA, leading to excessive secretion of inflammatory meditators and death and malfunction of nerve cells [3]. Excessive death of nerve cells or dysfunction can result in cognitive decline and memory loss, leading to the development of various neurodegenerative diseases such Alzheimer’s diseases, Parkinson’s diseases, or multiple sclerosis [4]. Therefore, since inflammatory agents produced by excessive inflammatory reactions can play a leading role in neurodegenerative diseases development, effective regulation of these abnormalities is suggested as an effective prevention method for these diseases.

*Curcuma longa* is a perennial herb belonging to the Zingiberaceae, originating in the southwestern India with a tropical climate. It is grown not only in India, but also in China and Myanmar, and is mainly grown in Jindo-gun, Jeollanam-do Province, Korea. The rhizome of *C. longa* is mainly used, and it is used in various ways in the food industry as herbal medicines, spices, beverages, tea, and food preservative, as well as used for coloring material [5]. It also has been utilized as a phyto-therapeutic for the treatment of variety of diseases including dyslipidemia, stomach disorders, arthritis, and hepatic diseases [6]. The main physiologically active components of *C. longa* are known as curcuminoids including curcumin, demethoxycurcumin, and bisdemethoxycurcumin [7], which have been reported to exhibit anti-bacterial, anti-HIV, antioxidant, anti-inflammatory, and anti-cancer effects [8]. Recently, it has been reported that hexane extract of *C. longa* exhibits anti-neuroinflammatory activity through inhibition of extracellular signal-regulated kinase (ERK) mitogen-activated protein kinase (MAPK) signaling pathway in microglial cell model that induced inflammation with lipopolysaccharide (LPS) [9].

According to Food Public Code provided by the Ministry of Food and Drug Safety in Korea, *C. longa* is included as “a material that can be used for a limited period of time in food,” which means that *C. longa* can be used only 50% of all raw materials when making certain foods. In order to resolve such restrictions, various extraction conditions for Korean *C. longa* should be established and various functions including safety should be continuously studied. Therefore, we studied the anti-neuroinflammatory effects of 50% ethanolic extract of *C. longa* grown in Korea on LPS-stimulated BV2 microglial cells.

## Methods

### Plant materials and preparation of CLE

The *C. longa* was harvested from the Jindo-gun, Jeollanam-do, Republic of Korea. The plant was authenticated by Yunji Lee, senior researcher of National Institute of Horticultural and Herbal Science, Rural Development Administration, Eumseong, Republic of Korea. A voucher specimen (MPS00) was deposited at the Herbarium of the Department of Herbal Crop Research, National Institute of Horticultural and Herbal Science, Rural Development Administration, Eumseong, Republic of Korea. The preparation of ethanolic extract of *C. longa* was performed as previously reported study using the same plant sample [10].

### Chemicals and reagents

The products and manufacturers used in this investigation are shown in Table 1.

**Table 1:** The product and manufacturers used in this investigation

Products	Manufacturers
RPMI1640 media	Gibco BRL Co.
Fetal bovine serum (FBS)	
Penicillin-streptomycin	
Phosphate-buffered saline (PBS)	
Trypsin-EDTA (TE)	
Lipopolysaccharide (LPS, O55:B5)	Sigma-Aldrich
Dimethyl sulfoxide (DMSO)	
3-(4,5-Dimethylathiazol-2yl)-2,5-diphenyltetrazoleum (MTT)	
Ammonium persulfate (APS)	
Tween-20	
PGE_2_ ELISA kit	ENZO Life Science, Inc.
IL-6 ELISA kit	R&D System
TNF-α ELISA kit	
RIPA lysis buffer	Thermo Fisher Scientific
Protease and phosphatase inhibitor cocktail	
NE-PER™ Nuclear and Cytoplasmic Extraction Reagents	
Nitrocellulose (NC) membrane	Bio-Rad Laboratories, Inc.
Bis-acrylamide solution	
Tris-HCl	
Tetramethylethylenediamine (TEMED)	
Tris-glycine SDS buffer	GenDepot
Tris-glycine native buffer	
Sodium dodecyl sulfate (SDS)	
Tris-buffered saline (TBS)	
ECL solution	
Skimmed milk powder	BD Biosciences
Anti-iNOS	Cell Signaling Technology Inc.
Anti-COX-2	
Anti-IкB-α	
Anti-p-IкB-α	
Anti-p65	
Anti-p-ERK	
Anti-ERK	
Anti-p-JNK	
Anti-JNK	
Anti-p-p38	
Anti-p38	
Anti-HO-1	
Anti-Nrf2	
Anti-β-actin	Santa Cruz Biotechnology Inc.
Anti-PCNA	
Secondary antibodies	Merck Millipore Co.

### Cell culture

BV2 cells were maintained at 5 × 10^5^ cells/mL in dishes of 100 mm in diameter in RPMI1640 supplemented with 10% (v/v) heat-inactivated FBS, penicillin G (100 units/mL), streptomycin (100 µg/mL), and l-glutamine (2 mM), and cultured at 37 ℃ in a humidified atmosphere containing 5% CO_2_.

### MTT assay for cell viability

BV2 cells were seeded in a 96-well plate, and were treated with CLE at various concentrations of 12.5 to 200 µg/mL for 24 h. MTT assay was performed to determine BV2 cell viability based on the previously reported protocol [11]. The absorbance was measured at 540 nm wavelength using Multiskan Microplate Reader (ThermoFisher). The optical density of the formazan solution from the control group (untreated group) was considered to indicate 100% viability.

### Determination of nitrite (NO production)

BV2 cells were seeded in a 24-well plate, pre-treated with CLE for 3 h, and stimulated with LPS (1 µg/mL) for 24 h. The level of NO production was estimated by measuring the nitrite concentration in the culture medium as described in previous report [11]. The nitrite concentration was determined using Multiskan Microplate Reader (ThermoFisher) with 540 nm wavelength.

### Assays for PGE_2_, IL-1β, IL-6 and TNF-α

BV2 cells were plated in 24-well plate, and pre-treated with CLE for 3 h, and stimulated with LPS (1 µg/mL) for 24 h. The supernatants were collected to determine the levels of PGE_2_ using ELISA kit from ENZO Life Science Inc. (Farmingdale, NY), and IL-1β, IL-6 and TNF-α using ELISA kit from R&D systems Inc. (Minneapolis, MN). The assays were performed according to the manufacturer’s instructions, and three independent replicates were performed.

### Quantitative real-time reverse transcription polymerase chain reaction (qRT-PCR)

BV2 cells were plated in 6-well plate, and pre-treated with CLE for 3 h, and stimulated with LPS (1 µg/mL) for 6 h. The detailed protocols of qRT-PCR were referenced in previous study [12]. The sequences of primers used in this experiment are shown in Table 2.

**Table 2:** Primers used for qPCR

Gene	Forward primer (5′ → 3′)	Reverse primer (3′→ 5′)
IL-1β	AATTGGTCATAGCCCGCACT	AAGCAATGTGCTGGTGCTTC
IL-6	ACTTCACAAGTCGGAGGCTT	TGCAAGTGCATCATCGTTGT
TNF-α	CCAGACCCTCACACTCACAA	ACAAGGTACAACCCATCGGC
GAPDH	TTCACCACCATGGAGAAGGC	GGCATGGACTGTGGTCATGA

### Western blot analysis

The proteins including iNOS, COX-2, NF-κB-related proteins, MAPK-related proteins, HO-1, and Nrf2 were detected by a Western blot analysis. The detailed protocols of Western blot analysis were referenced in previous study [11]. BV2 cells were pre-treated with CLE or SnPP, and stimulated with LPS (1 µg/mL). Cells were pelleted by centrifugation, were washed with PBS, and lysed with RIPA lysis buffer. The protein concentrations were measured using Bradford protein assay (Bio-Rad, CA, USA) and normalized to certify equal amount of were loaded. The equal mass (30 µg) of protein was resolved at 7.5 or 12% sodium dodecyl sulfate-polyacrylamide gel electrophoresis (SDS-PAGE). Then, proteins were transferred to NC membranes, and membranes were incubated with TBS-T with 5% skimmed milk for 1 h at 4 ℃. After that, membranes were probed with primary antibodies and incubated at 4 ℃ for 90 min or overnight. The membranes were washed with TBS-T, and the secondary antibodies were incubated for detecting primary antibodies. The protein bands were reacted with chemiluminescence reagent (GenDepot) for protein detection.

### Preparation of cytosolic and nuclear extracts

Nuclear and cytosolic extracts of the cells were obtained using the NE-PER™ Nuclear and Cytoplasmic Extraction Reagents. BV2 cells were pre-treated with CLE and stimulated with LPS (1 µg/mL) for 1 h. Cells were harvested by centrifugation, washed with PBS, and lysed with cytoplasmic extraction reagent which only decompose the cytoplasmic membrane without affecting nuclear envelop. After centrifugation, the supernatants which were used for cytoplasmic fraction were transferred to chilled tube, and the insoluble fraction (pellet) was lysed with nuclear extraction reagent. After centrifugation was completed, the supernatant was taken and transferred to chilled tube for use as a nuclear fraction.

### Statistical analysis

The data are the mean ± standard deviation (S.D) of at least three independent experiments. One-way analysis of the variance and subsequent Tukey’s multiple comparison tests were employed to compare three or more groups. All the statistical analyses were performed using GraphPad Prism software (version 3.03, GraphPad Software Inc., USA).

## Results

### The effect of CLE on the viability of BV2 microglial cells

**Fig. 1:**
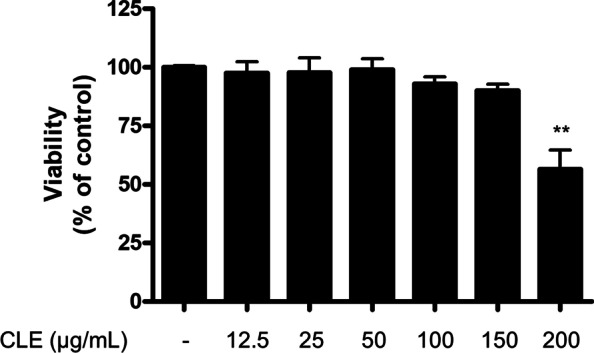
The effect of CLE on BV2 cell viability. Cells were treated with indicated concentrations of CLE for 24 h. The viability was determined by MTT assay

To evaluate the cytotoxicity of CLE, we determined the cell viability of BV2 cells following treatment with CLE for 24 h at concentrations ranging from 12.5 to 200 µg/mL. An MTT assay was conducted to determine optical density. The results of MTT assay showed that CLE at 12.5–150 µg/mL concentration had no effect of BV2 cell viability (Fig. 1).

### CLE inhibited the production of NO and PGE_2_ and the expression of iNOS and COX-2 proteins in LPS-induced BV2 microglial cells

**Fig. 2:**
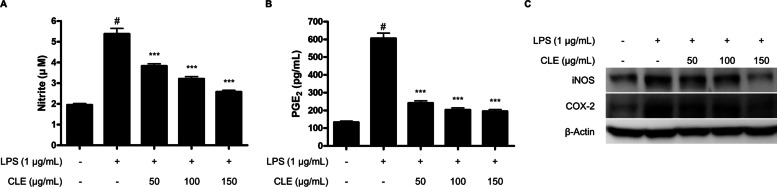
The effect of CLE on the production of NO and PGE_2_ (**A**, **B**), and the overexpression of iNOS and COX-2 (**C**) in LPS-induced BV2 microglial cells. Cells were pretreated with/without the indicated concentrations of CLE for 3 h and them stimulated with LPS (1 µg/mL) for 24 h. **A**, **B** The concentration of NO and PGE_2_ was determined by Griess assay and ELISA, respectively. Data represent the mean values of three independent experiments ± SD. ^#^*p* < 0.001 compared to the control group; **p < 0.01, and ***p < 0.001 compared to the LPS treated group. **C** The levels of iNOS and COX-2 proteins were determined by Western blot analysis. β-Actin was used as a loading control. Representative blots from three independent experiments are shown

The anti-inflammatory effects of CLE were initially evaluated through inhibition of NO and PGE_2_ production in LPS-induced BV2 microglial cells. Cells were pre-treated with CLE for 3 h with nontoxic concentration range of CLE, followed by stimulation with LPS (1 µg/mL) for 24 h. Treatment of BV2 cells with LPS triggered significant increase in the levels of NO and PGE_2_ compared to that in the untreated group. Pre-treatment with CLE significantly inhibited NO (Fig. 2A) and PGE_2_ (Fig. 2B) production by dose-dependent manner. These results urged us to investigate the inhibitory effects of CLE on the expression of iNOS and COX-2 proteins which produce NO and PGE_2_ as key pro-inflammatory mediators, respectively. CLE significantly suppressed the expression of iNOS and COX-2 proteins in dose-dependent manner (Fig. 2C).

### CLE inhibited the LPS-induced production of pro-inflammatory cytokines and the expression of those mRNA in BV2 cells

**Fig. 3:**
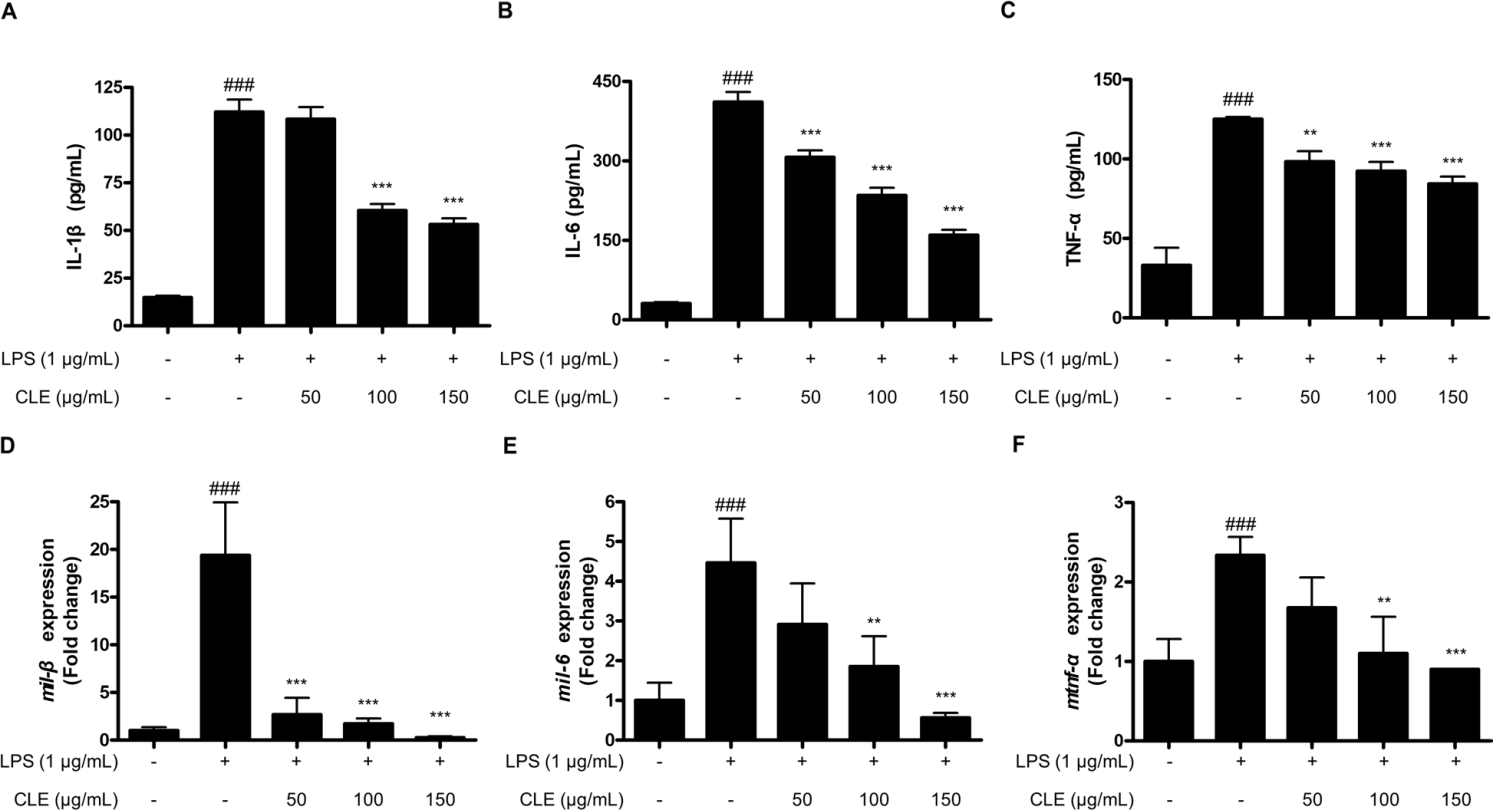
The effect of CLE on the LPS-induced production of pro-inflammatory cytokines (**A**-**C**) and their mRNA expression (**D**-**F**) in BV2 microglial cells. Cells were pre-treated with or without the indicated concentrations of CLE for 3 h, and then stimulated with LPS (1 µg/mL) for 24 h or 6 h. The levels of pro-inflammatory cytokines were quantified by ELISA, and mRNA expression was analyzed by qPCR. ^#^*p* < 0.001 compared to the control group; **p < 0.01, and ***p < 0.001 compared to the LPS treated group

The activated microglia could induce the excessive production of pro-inflammatory cytokines. To further examine the anti-inflammatory effects of CLE in LPS-induced BV2 microglial cells, the production of pro-inflammatory cytokines including IL-1β, IL-6 and TNF-α was estimated using ELISA kit. Cells were pre-treated with CLE for 3 h, and then incubated with LPS (1 µg/mL) for 24 h. The production of IL-1β, IL-6, and TNF-α levels increased in LPS-stimulated cells, and pre-treatment with CLE inhibited these responses dose-dependently (Fig. 3A-C). The mRNAs expression of IL-1β, IL-6, and TNF-α also suppressed by CLE treatment (Fig. 3D-F).

### CLE inhibited the LPS-induced activation of the NF-κB signaling pathway in BV2 microglial cells

**Fig. 4:**
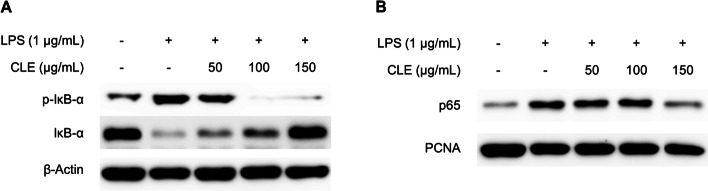
The effect of CLE on the activation of NF-κB signaling pathway in LPS-induced BV2 microglial cells. Cells were pre-treated with indicated concentrations of CLE for 3 h, and then stimulated with LPS (1 µg/mL) for 1 h. Nuclear and cytosolic extracts were isolated and the levels of p-IκB-α and IκB-α in the cytosolic fraction, and p65 in the nuclear faction were determined by Western blot analysis. β-Actin and PCNA were used as a loading control for cytoplasm and nuclear, respectively. Representative blots from three independent experiments are shown

Considering the inhibitory effects of CLE on the production of inflammatory mediators and the expression of inflammatory enzymes, we investigated the possible anti-inflammatory mechanisms underlying the inhibitory effects of CLE. BV2 cells were pre-treated with the indicated concentrations of CLE for 3 h, then stimulated with LPS (1 µg/mL) for 1 h, and we examined whether CLE regulates the nuclear factor kappa B (NF-κB) signaling pathway. LPS stimulation induced the phosphorylation and degradation of IκB-α, and pre-treatment with CLE inhibited these responses in a dose-dependent manner (Fig. 4A). As p65 is the major subunit of the NF-κB heterodimer, we next investigated the effect of CLE on the translocation of p65 subunit from the cytoplasm into the nucleus after being released from IκB-α. The levels of p65 subunit were increased in nucleus after LPS stimulation, whereas pre-treatment with CLE reversed the translocation of p65 (Fig. 4B).

### CLE inhibited the LPS-induced activation of the MAPK signaling pathways in BV2 microglial cells

**Fig. 5:**
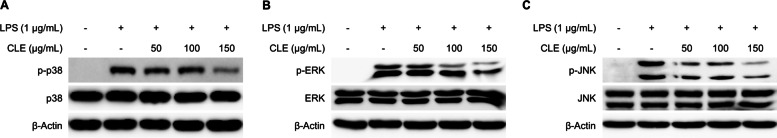
The effect of CLE on the activation of MAPK signaling pathway in LPS-induced BV2 microglial cells. Cells were pre-treated with indicated concentrations of CLE for 3 h, and then stimulated with LPS (1 µg/mL) for 1 h. The phosphorylated and total forms of p38, ERK, and JNK were determined by Western blot analysis. β-Actin and was used as a loading control. Representative blots from three independent experiments are shown

It was further investigated whether CLE affects the activation of MAPK signaling pathways. BV2 cells were pre-treated with the indicated concentrations of CLE for 3 h, and then stimulated with LPS (1 µg/mL) for 1 h. The phosphorylation of p38, ERK, and JNK MAPKs was increased after stimulation with LPS. Pre-treatment with CLE inhibited the phosphorylation of p38, ERK, and JNK MAPKs (Fig. 5).

### CLE induced HO-1 protein expression by activation of Nrf2 signaling pathway in BV2 microglial cells

**Fig. 6:**
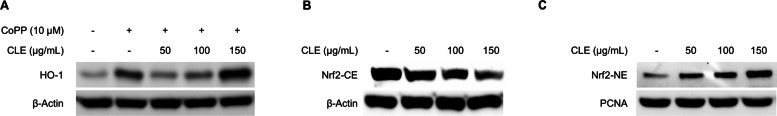
The effect of CLE on the HO-1 expression (**A**), and Nrf2 nuclear translocation (**B**, **C**) in BV2 cells. Cells were treated with CLE for 12 h at indicated concentrations. The levels of HO-1, cytosolic Nrf2, and nucleic Nrf2 were determined by Western blot analysis. β-Actin and PCNA were used as a loading control for cytoplasm and nuclear, respectively. Representative blots from three independent experiments are shown

**Fig. 7:**
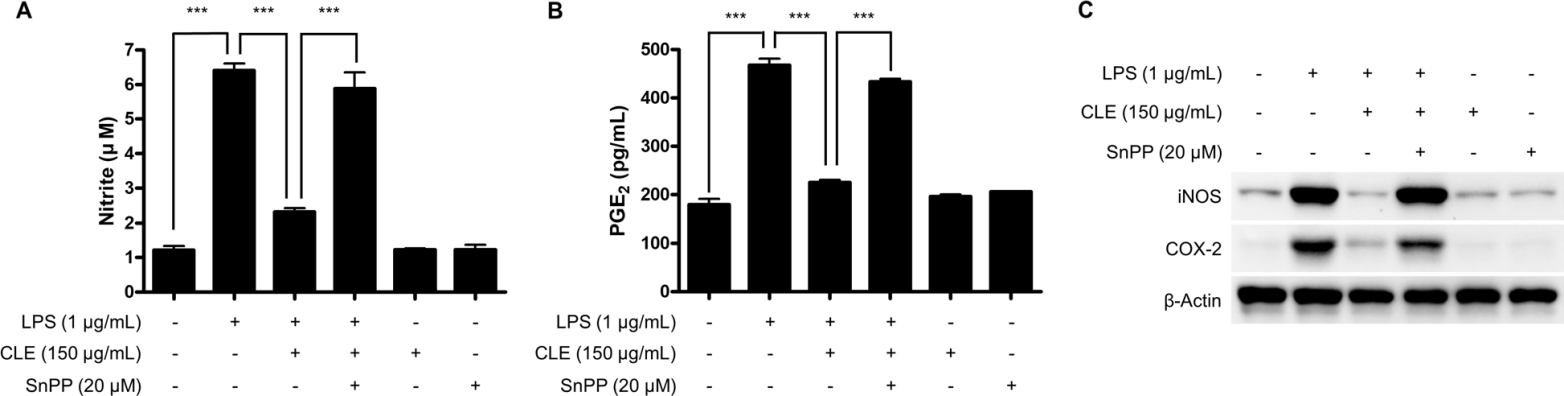
The inhibitory effect of SnPP on CLE-mediated reduction of NO, PGE_2_, iNOS, and COX-2. Cells were pre-treated with 150 µg/mL of CLE for 3 h with or without pre-treatment with SnPP for 1 h, and then stimulated with LPS (1 µg/mL) for 24 h. **A**, **B** The concentration of NO and PGE_2_ was determined by Griess assay and ELISA, respectively. Data represent the mean values of three independent experiments ± SD. ***p < 0.001. **C** The levels of iNOS and COX-2 proteins were determined by Western blot analysis. β-Actin was used as a loading control. Representative blots from three independent experiments are shown

To confirm whether CLE regulates HO-1 induction in BV2 microglial cells, we evaluated the expression of HO-1 protein by CLE in BV2 cells by Western blot analysis. Treatment with CLE for 12 h induced HO-1 protein expression (Fig. 6A), and this effect was regulated by CLE-mediated nuclear translocation of Nrf2 in BV2 cells (Fig. 6B, C). We also observed the correlation between HO-1 expression and anti-inflammatory effects by CLE. Cells were pre-treated with 150 µg/mL of CLE for 3 h with or without pre-treatment with SnPP for 1 h, and then stimulated with LPS (1 µg/mL) for 24 h. CLE decreased the LPS-induced production of NO and PGE_2_ and expression of iNOS and COX-2 proteins. The inhibition of HO-1 induction by SnPP offset the reduction of NO, PGE_2_, iNOS, and COX-2 by CLE, not by CLE and SnPP themselves (Fig. 7).

## Discussion

This investigation demonstrated that the 50% ethanolic extract of *Curcuma longa* (CLE) appeared to have anti-neuroinflammatory effect against LPS-induced inflammation in BV2 microglial cells. The anti-neuroinflammatory effect of CLE was associated with the inactivation of NF-κB and MAPK (p38, ERK, and JNK) signaling pathways, resulting in the inhibition of pro-inflammatory mediators including NO, PGE_2_, iNOS, COX-2, and pro-inflammatory cytokines such as including IL-1β, IL-6 and TNF-α. Moreover, CLE induced HO-1 proteins by activating Nrf2 signaling, and inhibiting HO-1 expression using SnPP reversed the inhibitory effects of CLE, indicating the anti-neuroinflammatory effect of CLE was mediated by HO-1/Nrf2 signaling pathway.

Neuroinflammation is generally characterized by the excessive production of pro-inflammatory mediators including NO, PGE_2_, iNOS, COX-2, and pro-inflammatory cytokines [13]. NO is catalyzed by the enzymatic activity of iNOS which converts L-arginine to NO and L-citrulline via the intermediate N-hydroxy-L-arginine [14], and PGE_2_ is synthesized from arachidonic acid (AA) by the enzymatic effect of COX and PGE synthases (PGES) [15]. Pro-inflammatory cytokines are small secreted proteins from various immune cells, and they play multiple roles in CNS function including the regulation of sleep, neuronal development, and inflammatory responses against bacterial and viral infections of either the brain or the periphery [16]. Therefore, the inactivation of these pro-inflammatory mediators could be one of the targets for treatment and prevention of neuroinflammatory diseases. This study sought to elucidate the anti-neuroinflammatory effects of CLE in LPS-induced BV2 microglial cells. The overproduction of NO/PGE_2_, and the protein expression of iNOS/COX-2 were inhibited by pre-treatment with CLE (Fig. 2). In addition, CLE decreased the overproduction of pro-inflammatory molecules and their mRNA expression including of IL-1β, IL-6, and TNF-α in LPS-induced BV2 microglial cells (Fig. 3).

NF-κB represents a family of inducible transcription factors, which regulates a large array of genes involved in different processes of the immune and inflammatory responses [17]. It is composed of five different members including p65 (RelA), RelB, c-Rel, NF-κB1 (p50/p105), and NF-κB2 (p52/p100), which mediates transcription of target genes by binding to a specific DNA element, κB enhancer, as various hetero- or homo-dimers [18]. NF-κB is activated via two major signaling pathways including the classical/canonical NF-κB pathway, and the alternative/non-canonical NF-κB pathway, and they are both important for regulating inflammatory responses [19, 20]. The canonical NF-κB pathway which are most extensively studied is regulated by the activation of a variety of cell surface receptors including IL-1 receptor, Toll-like receptors (TLRs), TNF receptor, as well as T-cell receptor and B-cell receptor [18], whereas the non-canonical NF-κB pathway can be triggered by a specific group of stimuli including ligands of subset of TNF-receptor superfamily members such as B cell activating factor (BAF), CD40, lymphotoxin β (LTβ) receptor, and receptor activator of NF-κB (RANK) [17]. NF-κB can be activated by a wide variety of stimuli including viruses, bacterial toxins, UV light, oxidative stresses, inflammatory stimuli, cytokines, carcinogens, tumor promoters, and various mitogens [21, 22], and it regulates the expression of inflammatory-related genes including iNOS, COX-2, lipoxygenase (LOX), cytokines, adhesion molecules, cell cycle regulatory molecules, and angiogenic factors [23]. Therefore, the inactivation of NF-κB is one of the important targets for the treatment of neuroinflammation. In this study, pre-treatment with CLE attenuated LPS-induced activation of NF-κB signaling pathway by inhibiting the phosphorylation and degradation of IκB-α, and the translocation into the nucleus of p65 subunit in BV2 microglial cells (Fig. 4).

MAPKs comprise a family of serine/threonine protein kinases that have been implicated in the regulation of key cellular processes including gene induction, cell survival/apoptosis, proliferation and differentiation as well as cellular stress and inflammatory responses [24]. MAPK signaling pathways are triggered by the activation of TLRs, toll-interleukin receptor (TIR), or the TNF receptor families by primary inflammatory stimuli and cytokines [25]. In mammals, MAPKs pathways consist of three major subunits including p38, ERK, and JNK [26]. ERK1 and ERK2 which are homologous isoform and expressed in nearly all tissues are activated by MAPK kinase (MKK) and MKK2 and have important role in proliferation, cell death, cytoskeletal remodeling, and regulating cell shape and motility [25, 26]. JNKs consist of at least ten isoforms derived from alternatively spliced mRNAs of three genes including JNK1, JNK2, and JNK3 [26]. They are also called as stress-activated kinases (SAPKs) because of their activation in response to cell stress [27]. The activation of JNK is regulated by MKK4 and MKK7, and is important for survival of cells and replication of viruses [25]. Four p38 isoforms (p38α, p38β, p38γ, and p38δ) are found in mammals. p38α and p38β isoforms are expressed in most of tissues, whereas p38γ, and p38δ isoforms are expressed in kidney, skin and muscle cells [28]. They are activated by MKK3, MKK4, and MKK6 [25], and the activation of p38 isoforms leads to the phosphorylation of transcription factors which regulate pro-inflammatory mediators [29]. The inhibition of MAPK signaling pathway emerge as attractive anti-inflammatory agents, because they can reduce reducing the synthesis of inflammation mediators at multiple levels and are effective in blocking inflammatory cytokine signaling [30]. Our results showed that pre-treatment with CLE inhibited LPS-induced activation of p38, ERK, and JNK MAPKs by inhibiting the phosphorylation of them in BV2 microglial cells (Fig. 5).

HO has three distinct isoforms of HO including the only inducible form HO-1, which is the only inducible form and is known as heat-shock protein 32 (Hsp-32), and constitutively expressed HO-2, and HO-3 [31, 32]. In particular, HO-1 is a detoxifying phase II anti-oxidant enzyme, which is up-regulated in various pathological conditions including cellular stresses and stimuli including ischemia, hypoxia, oxidative stress, and inflammatory cytokines [33, 34]. Under oxidative injury and inflammatory conditions, HO-1 acts the rate-limiting enzyme in the catabolism of heme conversing into carbon monoxide (CO), ferrous ion (Fe^2+^), and biliverdin, which act as anti-oxidant and anti-inflammatory mediators and reported alleviating extent of oxidative stress and related disorders [33, 35]. Moreover, it has also been demonstrated that HO-1 expression is up-regulated by anti-inflammatory cytokines [36], indicating that HO-1 may be a therapeutic target in neurodegenerative diseases and brain infection [37]. HO-1 expression is controlled by the Nrf2 signaling pathway. Nrf2 is a member of the cap-n-collar (CNC) transcription factor family of basic leucine zipper proteins [38]. It is a crucial factor in regulation of cellular redox homeostasis, oxidative stress and immune inflammation [39, 40]. In resting state, Nrf2 is bound to the endogenous inhibitor Kelch-like ECH-associated protein 1 (Keap 1) in the cytoplasm, which induces ubiquitination and proteasomal degradation of Nrf2 [40, 41]. Under oxidative stress or inflammatory conditions, Nrf2 dissociates from Keap1, translocates into the nucleus, forms a heterodimer with the small Maf proteins that recognize and binds to antioxidant response elements (ARE) in the promoter site of phase II detoxifying enzymes and cytoprotective genes including including HO-1, NAD(P)H quinone oxidoreductase 1 (NQO1), peroxiredoxin (PRX), thioredoxin (Trx), glutathione S-transferase (GST), and glutathione peroxidase (GPx) [40, 42]. In addition, Nrf2-ARE binding also regulates the expression of genes related to pro- and anti-inflammatory enzymes including iNOS and COX-2 [43]. Various kinds of natural products have been reported to up-regulate HO-1 expression by activating Nrf2 to bind with the ARE such as berberine from *Coptidis chinensis*, 7,8-dihydroxyflavone, or tryptanthrin in astrocytes, myoblast, and microglial cells [35, 44, 45]. In the present study, CLE induced HO-1 expression, as well as the accumulation of Nrf2 in the nucleus. In addition, pre-treatment with SnPP, a HO-1 inhibitor, abolished the CLE-induced inhibition of secretion of NO and PGE_2_ as well as the expression of iNOS and COX-2 proteins. Taken together, these results indicate that CLE-induced activation of HO-1/Nrf2 signaling pathway plays a crucial role in downregulating neuroinflammatory responses.

As it is known, *C. longa* is composed of various compounds including diarylheptanoids (including curcuminoids), diarylpentanoids, monoterpenes, sesquiterpenes, diterpenes, triterpenoids, alkaloid, and sterols, of which curcuminoids are the most abundant [46]. Therefore, in order to analyze the content of curcuminoids that are the most abundant and exhibit various physiological activities among the components contained in *C. longa*, we established a quantitative analysis method using the ethanolic extract of *C. longa* grown in Korea and verified the content of curcuminoids in previous study [10]. The ethanolic extract of *C. longa* is an optimal condition for analyzing the content of curcuminoids, and its physiological activities may be due to curcuminoids. However, since *C. longa* also contains other compounds such as monoterpenes or sesquiterpenes, it is necessary to further verification which compound is responsible for anti-neuroinflammatory effects of CLE.

In this investigation, setting the maximum concentration of CLE to 150 µg/mL could be considered an appropriate measure. First of all, our study evaluated the cytotoxicity of CLE in BV2 cells as shown in Fig. 1, and the results confirmed that it was toxic at 200 µg/mL but not at 150 µg/mL. Second, in the previous report confirming the anti-oxidant effect in BV2 microglial cells using the hexane extract of *C. longa*, the experiment was conducted by setting the maximum concentration of the hexane extract of *C. longa* to 500 µg/mL [9]. Finally, other studies that examined the anti-neuroinflammatory effects in BV2 microglial cells using the extract of other natural products, the concentration much higher than 150 µg/mL used in this study was set as the highest concentration [47, 48]. Therefore, the 150 µg/mL of CLE used in this investigation is considered to be sufficient to examine the anti-neuroinflammatory activity.

## Conclusion

**Fig. 8:**
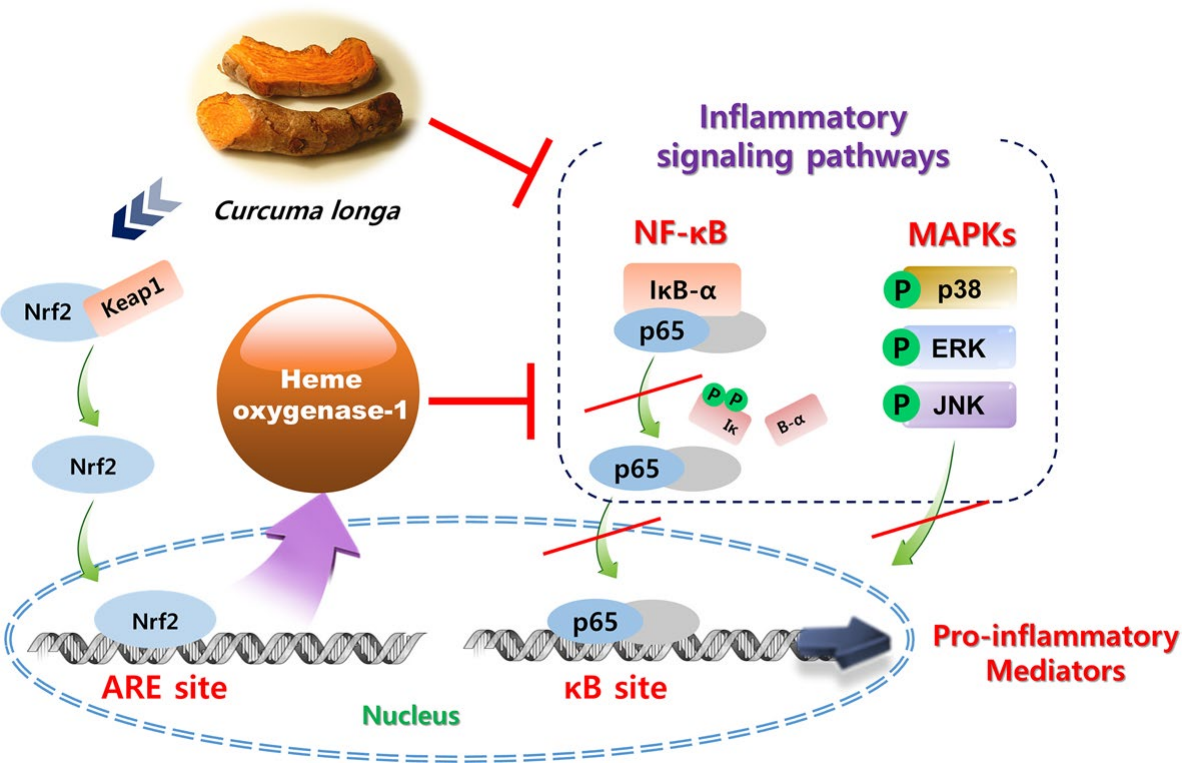
Scheme of the molecular mechanisms of the anti-neuroinflammatory effects of CLE

The present study demonstrated that CLE reduced the LPS-induced overproduction of inflammatory mediators triggered by NF-κB, p38 MAPK, ERK MAPK, and JNK MAPK through activating HO-1/Nrf2 signaling pathway in BV2 microglial cells (Fig. 8). Our results suggest that CLE represents potential anti-inflammatory candidates for further investigations for the treatment of the neurodegenerative diseases.

## Supplementary Information

**Additional file 1.**

## Data Availability

The datasets used and/or analyzed during the current study are available from the corresponding author on reasonable request.

## Abbreviations

AA: Arachidonic acid
APS: Ammonium persulfate
ARE: Antioxidant response element
BAF: B cell activating factor
CNC: Cap-n-collar
CNS: Central nervous system
CO: Carbon monoxide
COX-2: Cyclooxygenase-2
DEPC: Diethylpyrocarbonate
DMSO: Dimethyl sulfoxide
ECL: Enhanced chemiluminescence
ELISA: Enzyme-linked immunosorbent assay
ERK: Extracellular signal-regulated kinase
FBS: Fetal bovine serum
GAPDH: Glyceraldehyde-3-phosphate dehydrogenase
GPx: Glutahione peroxidase
GST: Glutathione S-transferase
HO-1: Heme-oxygenase-1
Hsp: Heat-shock protein
IL: Interleukin
iNOS: Inducible nitric oxide synthase
IκB: Inhibitor kappa B
JNK: c-Jun N-terminal kinase
Keap1: Kelch-like ECH-associated protein 1
LOX: Lipoxygenase
LPS: Lipopolysaccharide
LTβ: Lymphotoxinβ
MAPK: Mitogen-activated protein kinase
MKK: MAPK kinase
MTT: 3-(4,5-Dimethylathiazol-2yl)-2,5-diphenyltetrazoleum
NC membrane: Nitrocellulose membrane
NF-κB: Nuclear factor kappa B
NO: Nitric oxide
NQO1: NAD(P)H quinone oxidoreductase 1
Nrf2: Nuclear factor erythroid-2-related factor 2
PBS: Phosphate-buffered saline
PCNA: Proliferating cell nuclear antigen
PGE_2_: Prostaglandin E2
PGES: Prostaglandin E synthase
PRX: Peroxiredoxin
RANK: Receptor activator of NF-κB
RIPA: Radioimmunoprecipitation
SAPK: Stress-activated kinase
SDS: Sodium dodecyl sulfate
SnPP: Tin protoporphyrin IX
TBS: Tris-buffered saline
TE: Trypsin-ethylene-diamine-tetra acetic acid
TEMED: Tetra methyl ethylene diamine
TIR: Toll-interleukin receptor
TLR: Toll-like receptor
TNF: Tumor necrosis factor
Trx: Thioredoxin
