# Supplementary material for: The ethanolic extract of Curcuma longa grown in Korea exhibits anti-neuroinflammatory effects by activating of nuclear transcription factor erythroid-2-related factor 2/heme oxygenase-1 signaling pathway
Source: BMC Complement Med Ther. 2022 Dec 30;22:343. doi: 10.1186/s12906-022-03825-5 (PMC9804997; doi:10.1186/s12906-022-03825-5)

**Supplementary Information**

Journal: BMC Complementary Medicine and Therapies

Title: “The ethanolic extract of *Curcuma longa* grown in Korea exhibits anti-neuroinflammatory effects by activating of nuclear transcription factor erythroid-2-related factor 2/heme oxygenase-1 signaling pathway”

Kwan-Woo Kim^a^, Young-Seob Lee^a,*^, Dahye Yoon, Geum-Soog Kim, and Dae Young Lee^*^

Department of Herbal Crop Research, National Institute of Horticultural and Herbal Sciences, Rural Development Administration, Eumseong 27709, Republic of Korea

* Correspondence: [youngseoblee@korea.kr](mailto:youngseoblee@korea.kr); [dylee0809@gmail.com](mailto:dylee0809@gmail.com)

**Supplementary Figure 1.** Original Western blots for iNOS, COX-2 (A), and corresponding β-actin proteins (B) in LPS-induced BV2 microglial cells pre-treated with CLE.


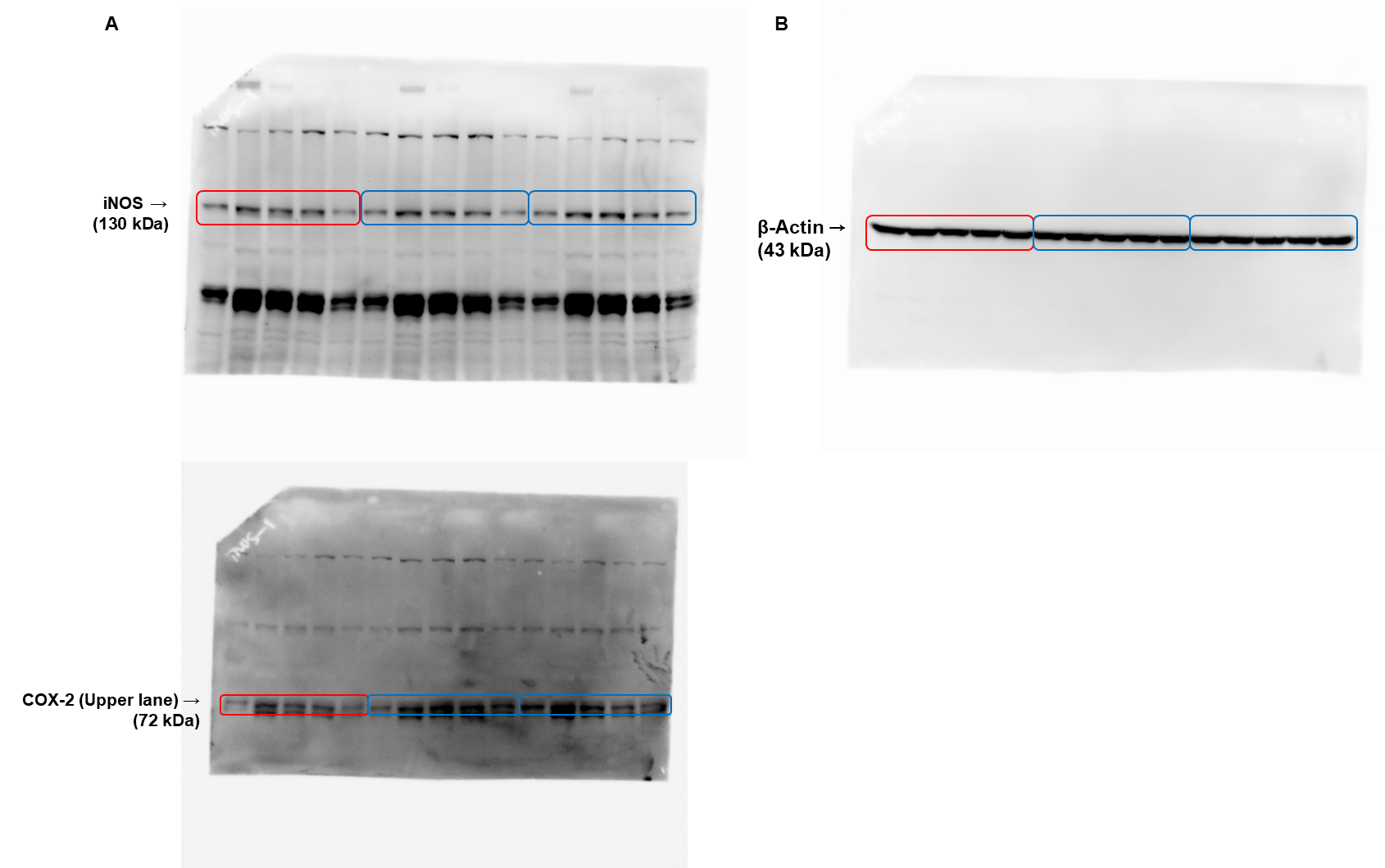


**Supplementary Figure 2.** Cropped version of iNOS, COX-2, and their corresponding β-actin proteins in LPS-induced BV2 microglial cells pre-treated with CLE.


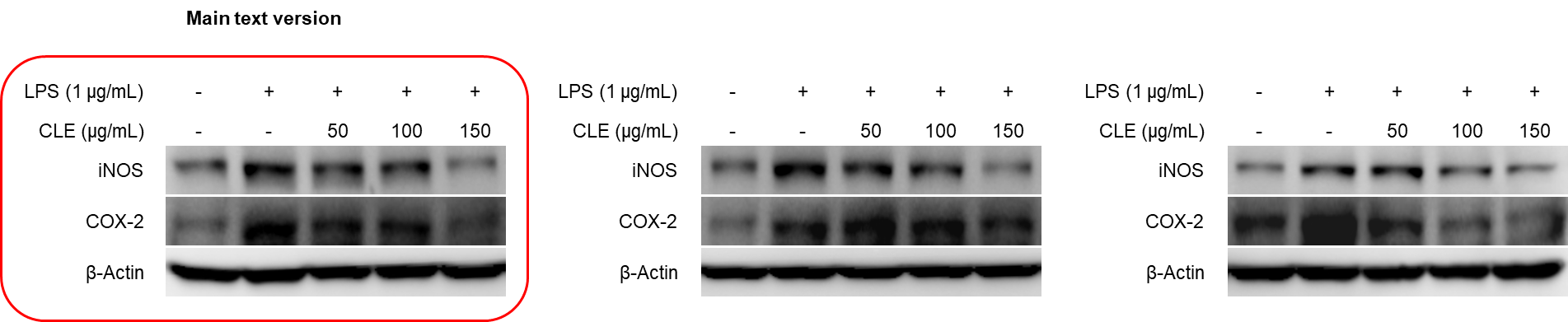


**Supplementary Figure 3.** Original Western blots for p65 (A, B), and corresponding PCNA proteins (C, D, E) in LPS-induced BV2 microglial cells pre-treated with CLE. “Other sample” means that when conducting experiments using CLE, an experiment using another sample was performed together. The images in which the membrane edges of the original blots are not clearly visible or appear to be cropped versions may be due to detection of protein in a short time because the proteins and primary antibodies interacted actively. The presented images are full-length, original, and unprocessed blots which were not cut prior to hybridization with antibodies during blotting.

**
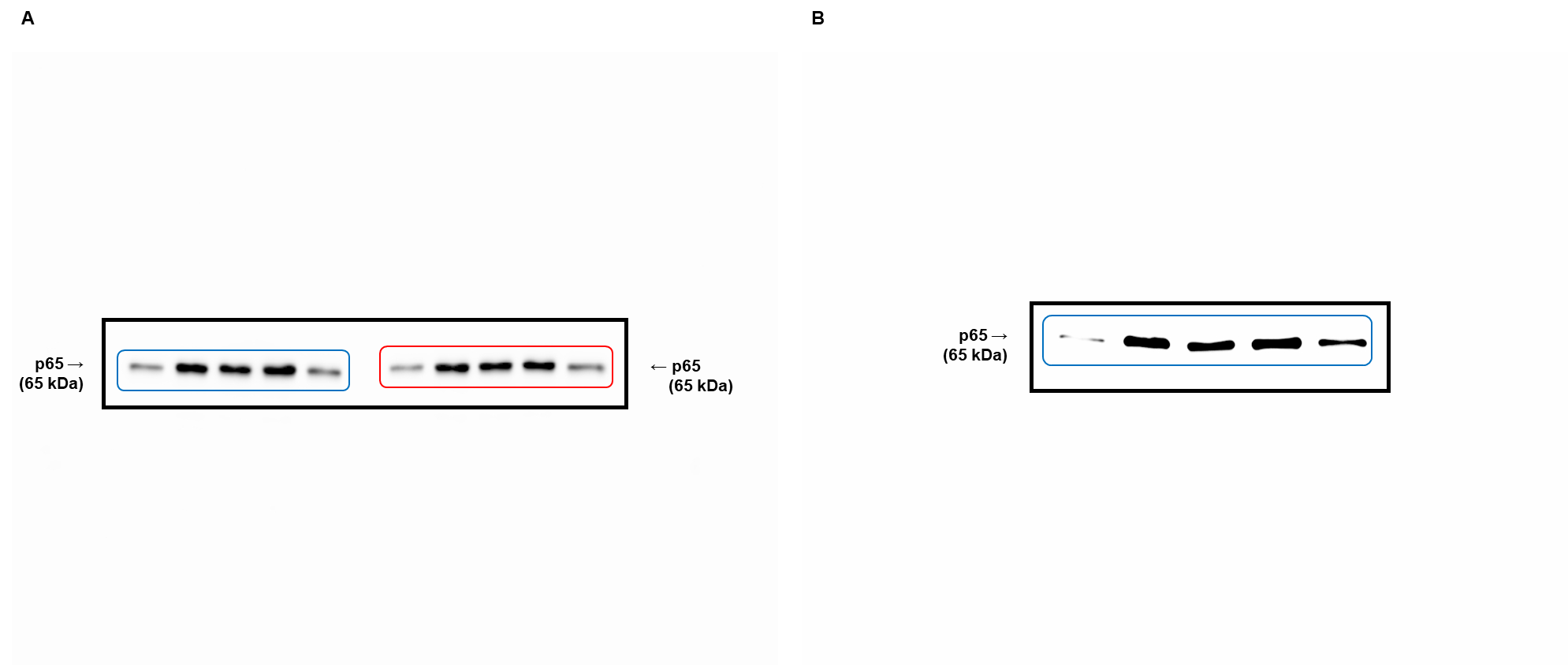
**

**
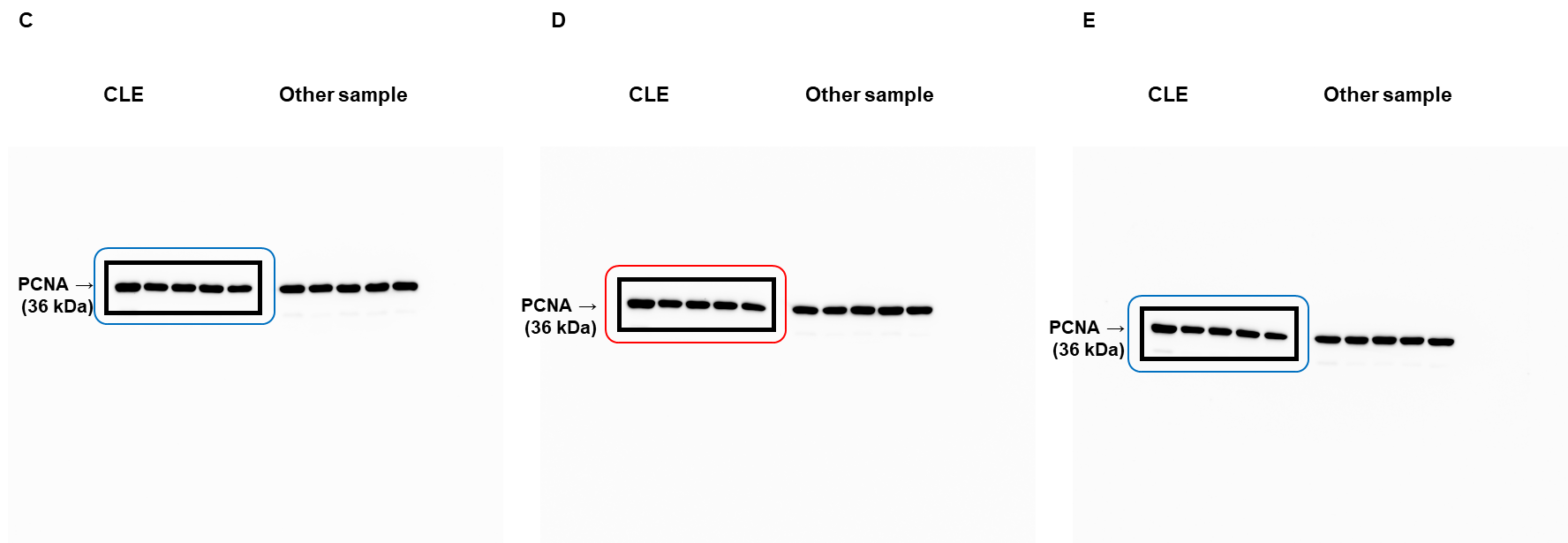
**

**Supplementary Figure 4.** Cropped version of p65 in nucleic fraction and their corresponding PCNA proteins in LPS-induced BV2 microglial cells pre-treated with CLE.


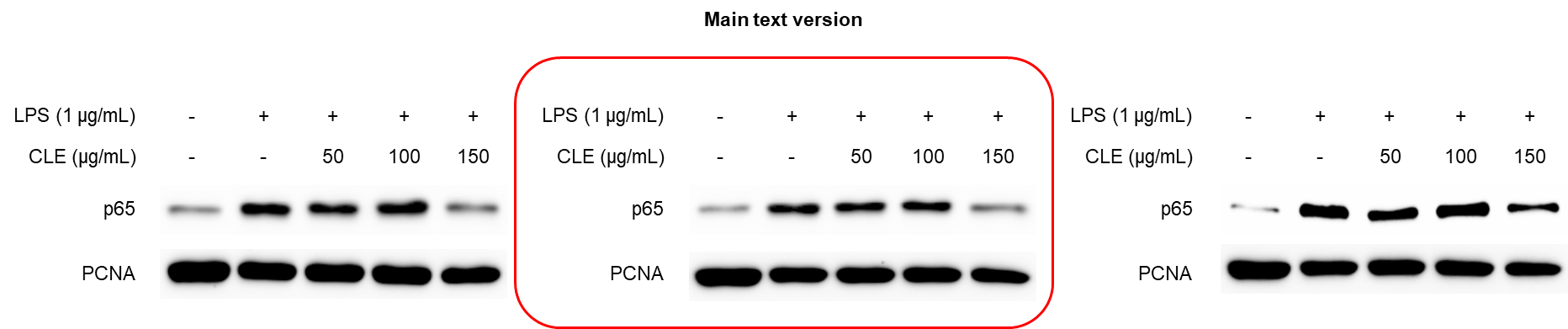


**Supplementary Figure 5.** Original Western blots for pIκB-α (A), IκB-α (B, C, D), and corresponding β-actin (E, F) proteins in LPS-induced BV2 microglial cells. “Other sample” means that when conducting experiments using CLE, an experiment using another sample was performed together. The images in which the membrane edges of the original blots are not clearly visible or appear to be cropped versions may be due to detection of protein in a short time because the proteins and primary antibodies interacted actively. The presented images are full-length, original, and unprocessed blots which were not cut prior to hybridization with antibodies during blotting.


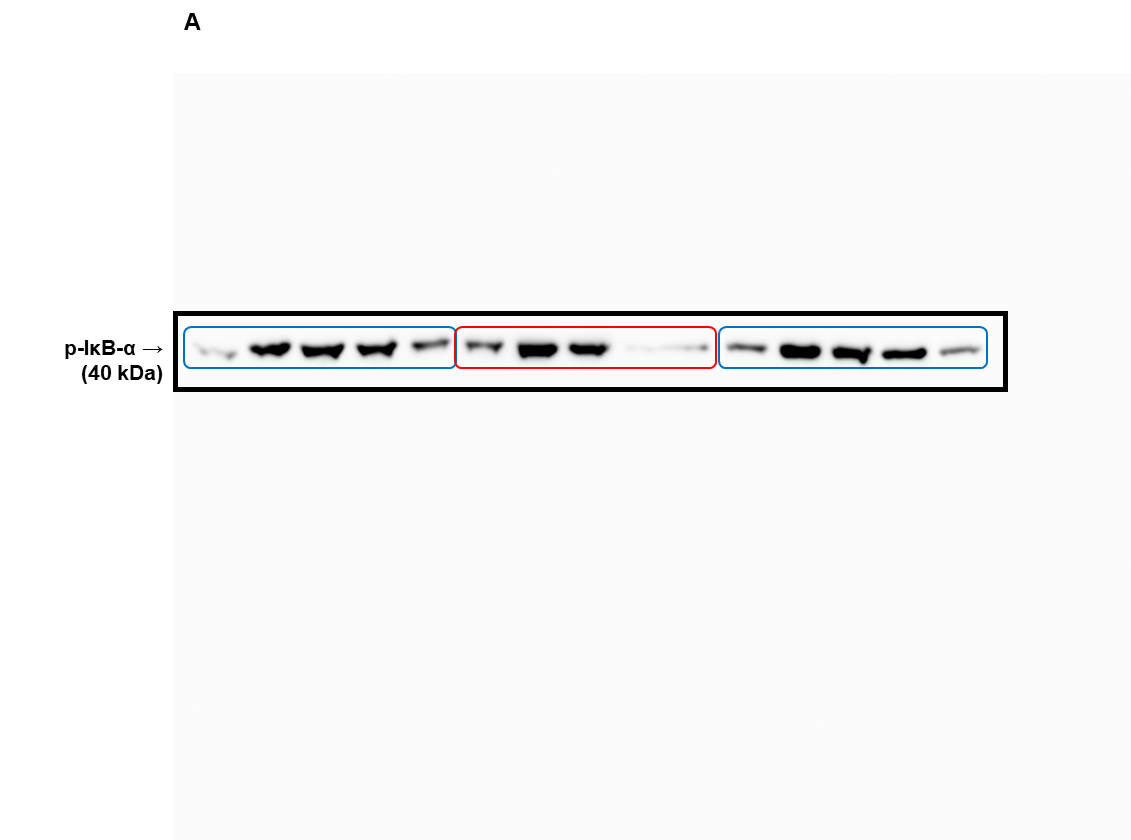


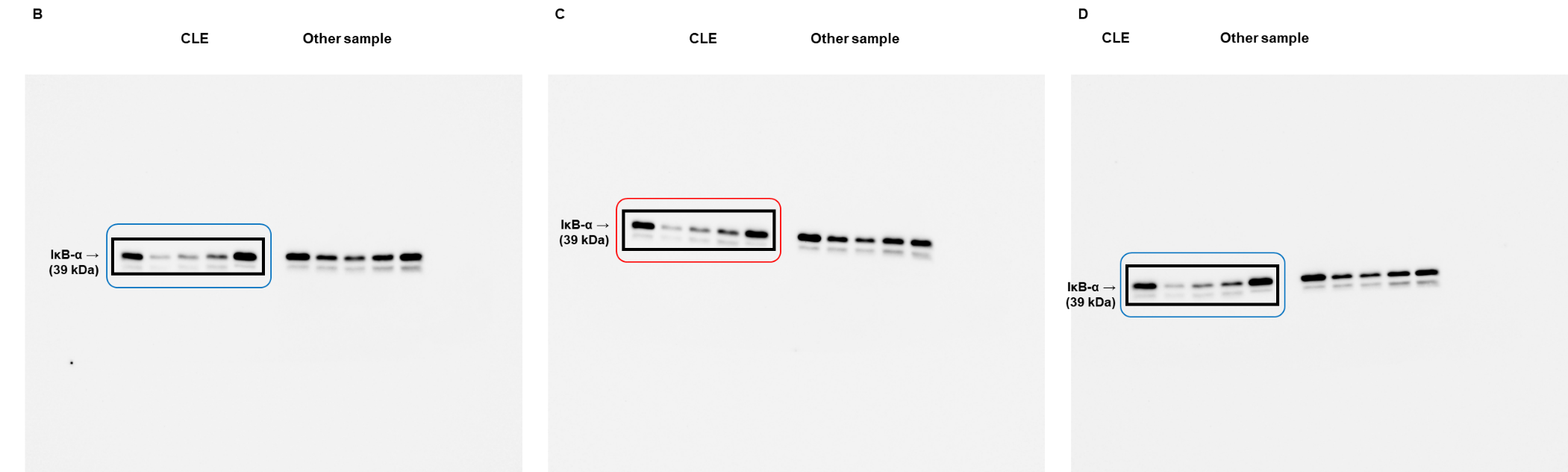


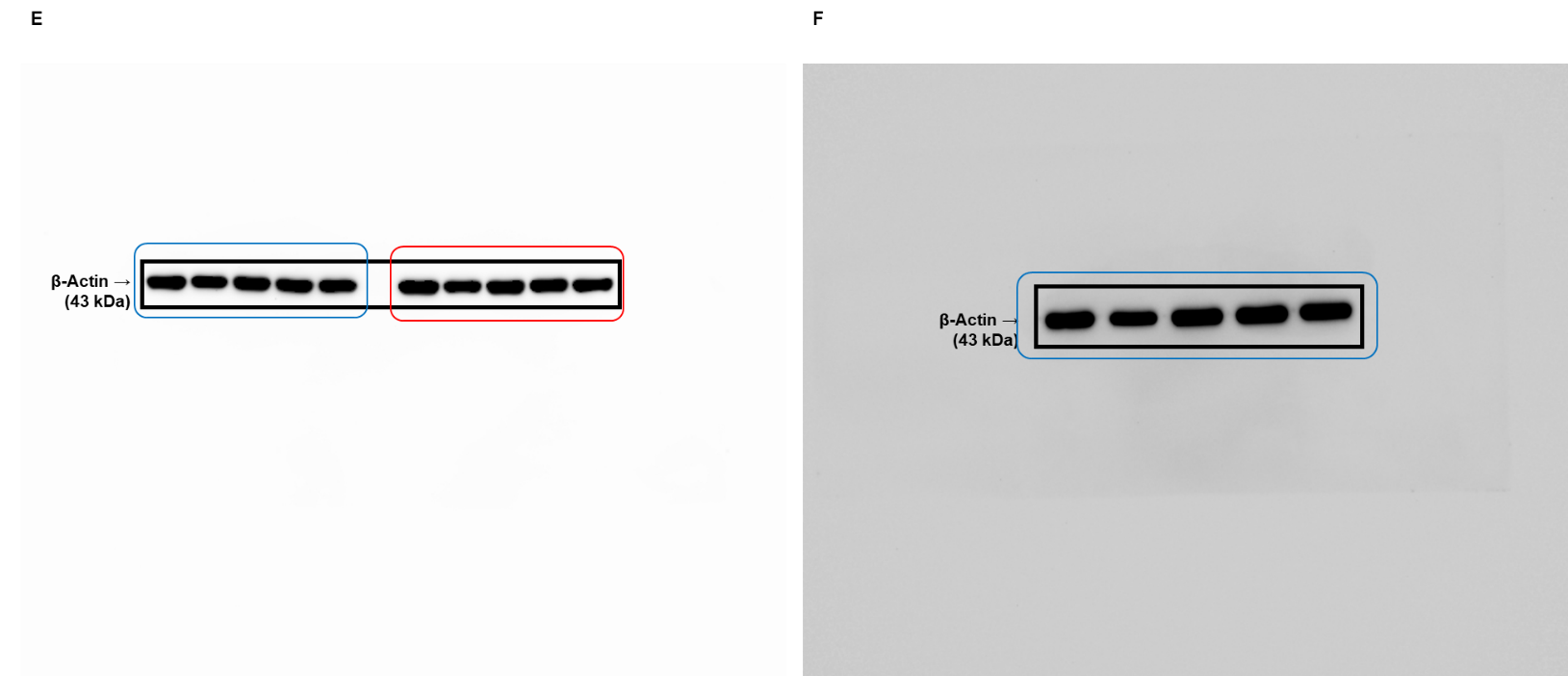


**Supplementary Figure 6.** Cropped version of pIκB-α, IκB-α, and their corresponding β-actin proteins in LPS-induced BV2 microglial cells pre-treated with CLE.


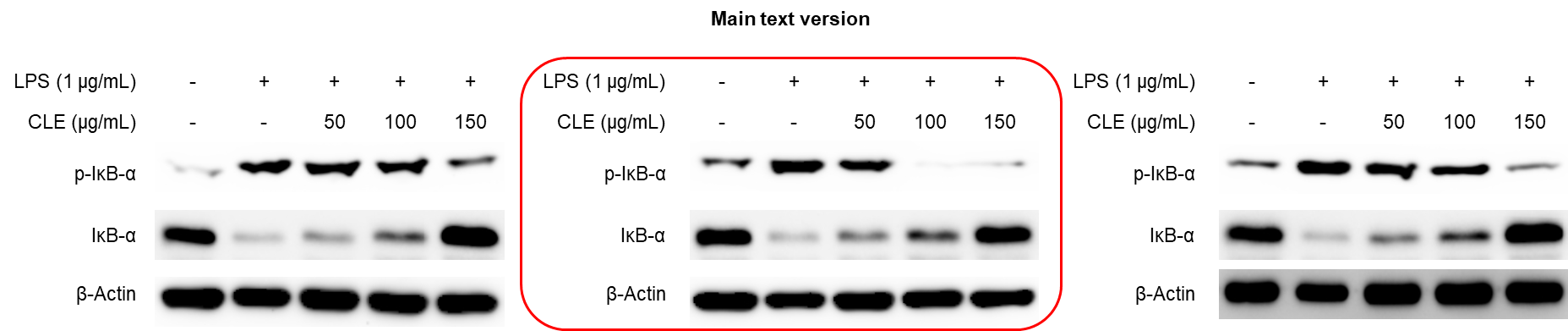


**Supplementary Figure 7.** Original Western blots for p-p38 (A), p38 (B), and corresponding β-actin proteins (C) in LPS-induced BV2 microglial cells. The images in which the membrane edges of the original blots are not clearly visible or appear to be cropped versions may be due to detection of protein in a short time because the proteins and primary antibodies interacted actively. The presented images are full-length, original, and unprocessed blots which were not cut prior to hybridization with antibodies during blotting.


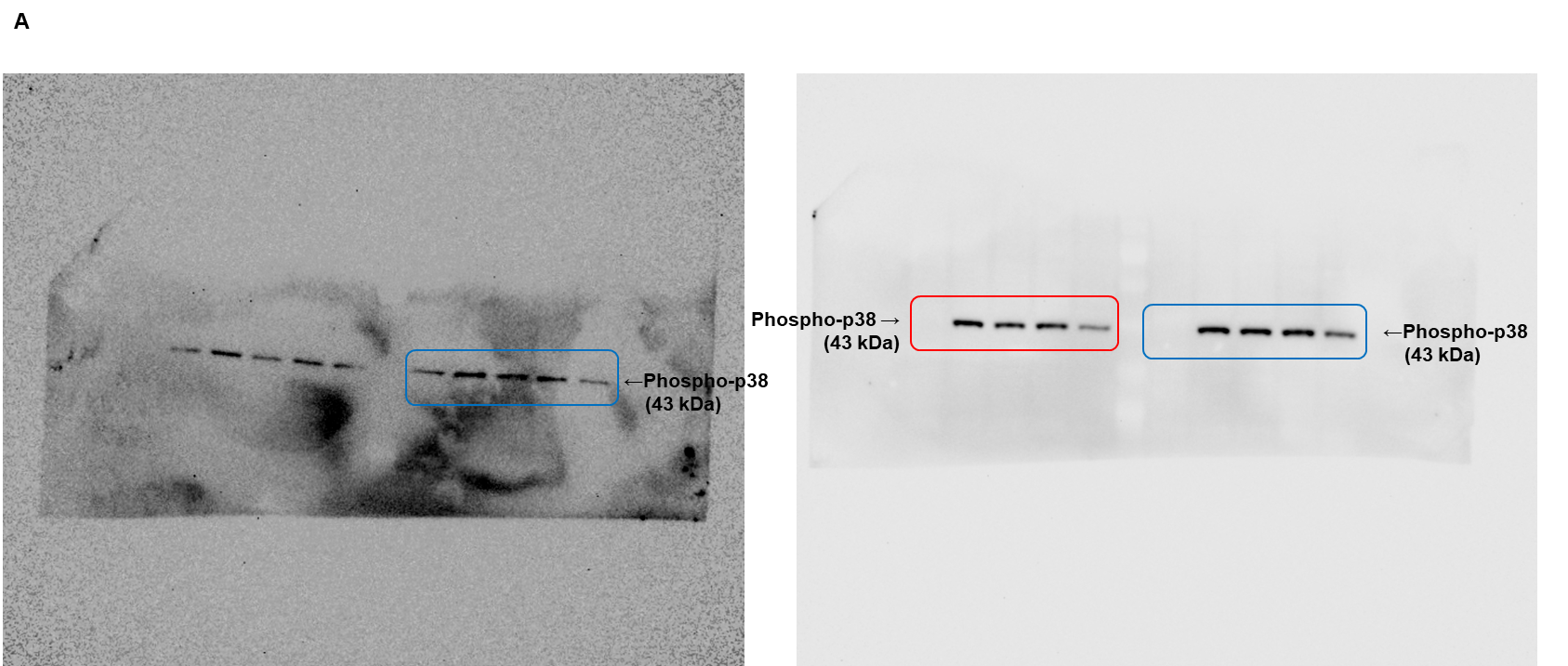


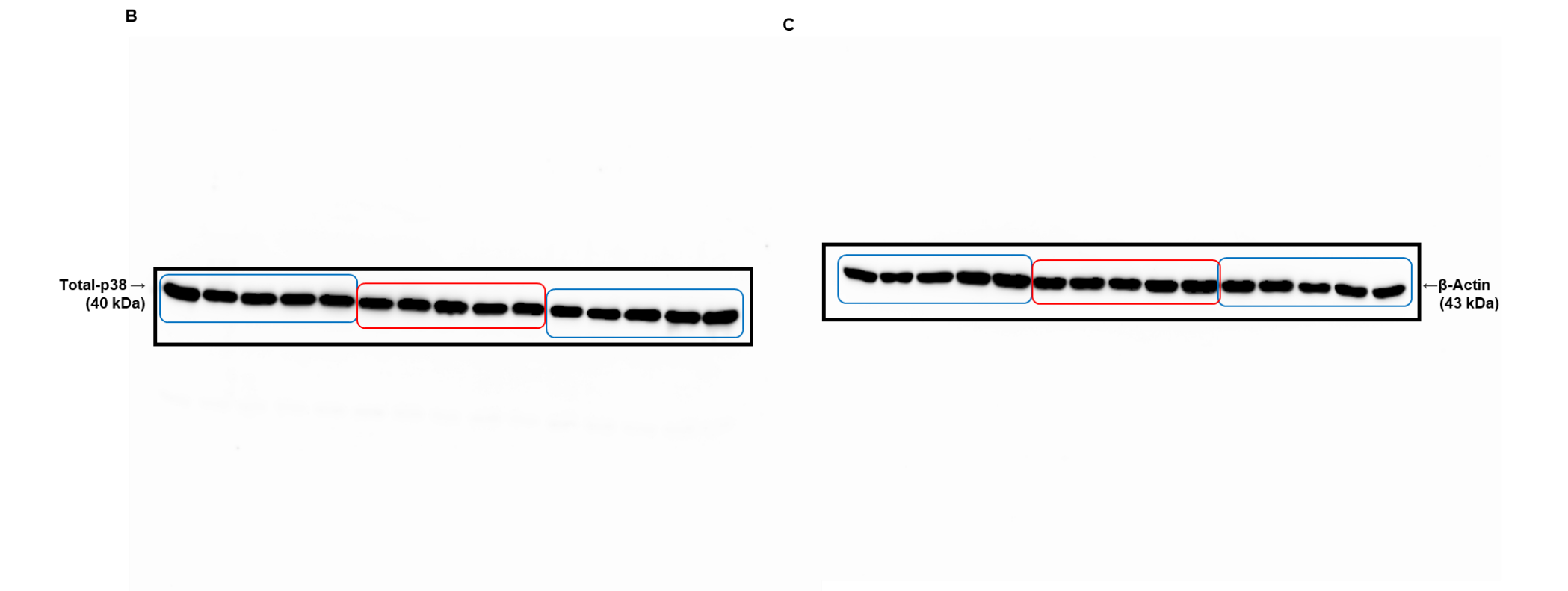


**Supplementary Figure 8.** Cropped version of p-p38, total p38, and their corresponding β-actin proteins in LPS-induced BV2 microglial cells pre-treated with CLE.


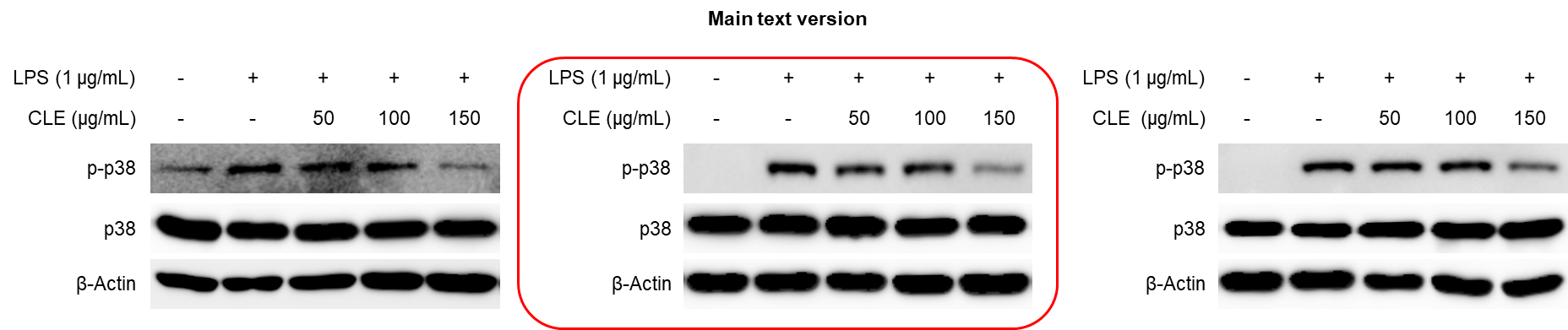


**Supplementary Figure 9.** Original Western blots for p-ERK (A, B), ERK (C, D), and corresponding β-actin proteins (E) in LPS-induced BV2 microglial cells. The images in which the membrane edges of the original blots are not clearly visible or appear to be cropped versions may be due to detection of protein in a short time because the proteins and primary antibodies interacted actively. The presented images are full-length, original, and unprocessed blots which were not cut prior to hybridization with antibodies during blotting.


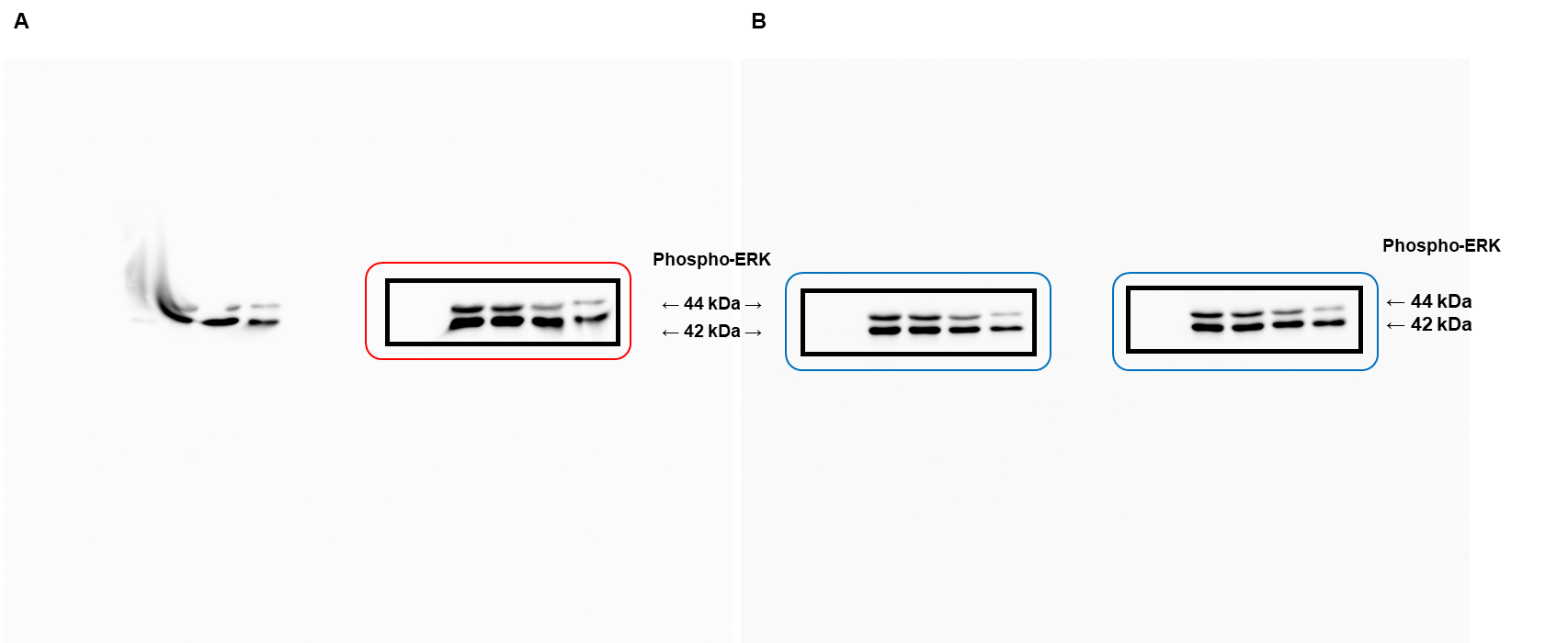


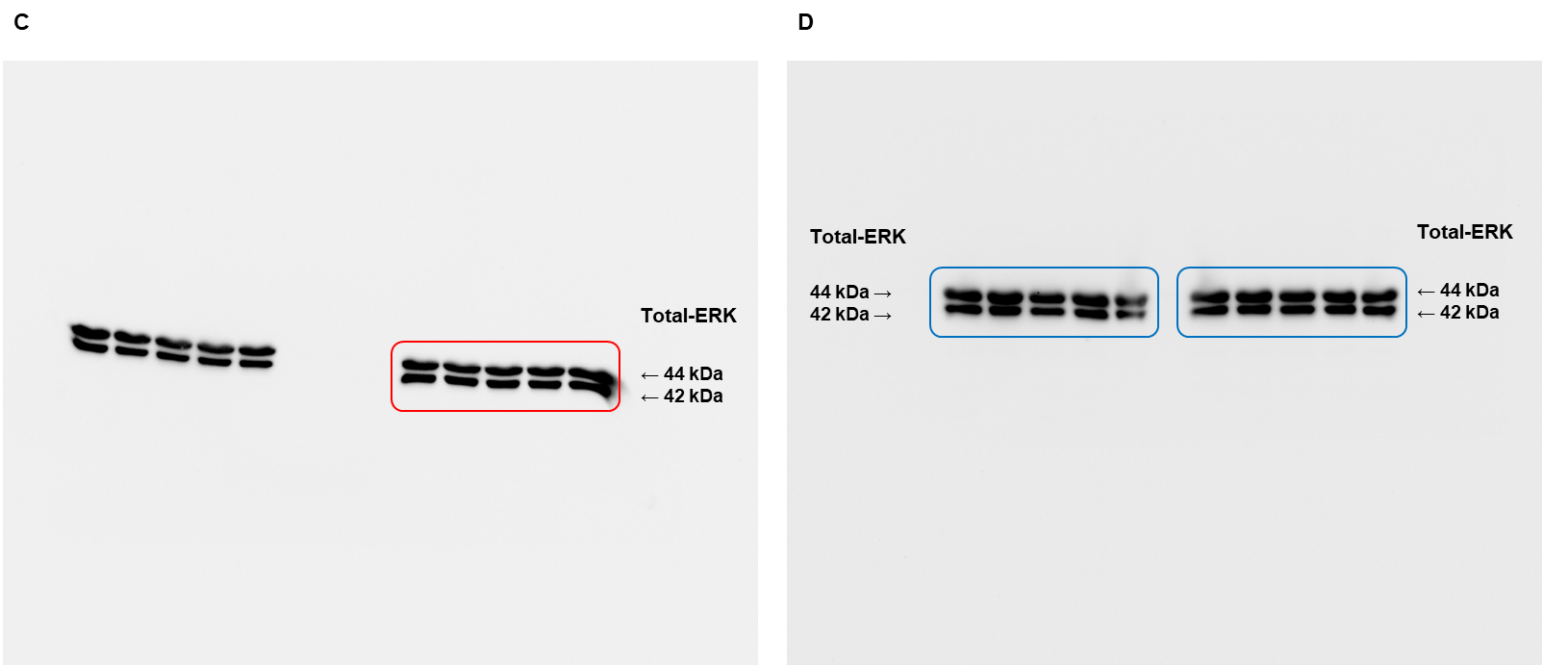


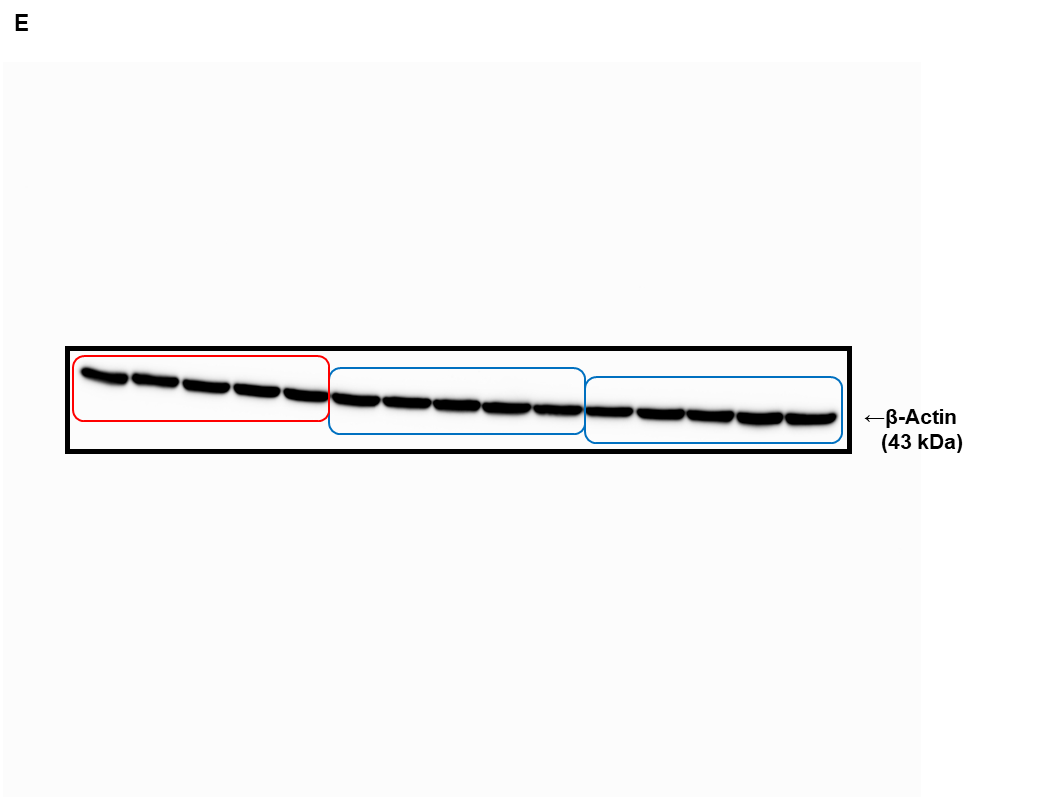


**Supplementary Figure 10.** Cropped version of p-ERK, total ERK, and their corresponding β-actin proteins in LPS-induced BV2 microglial cells pre-treated with CLE.


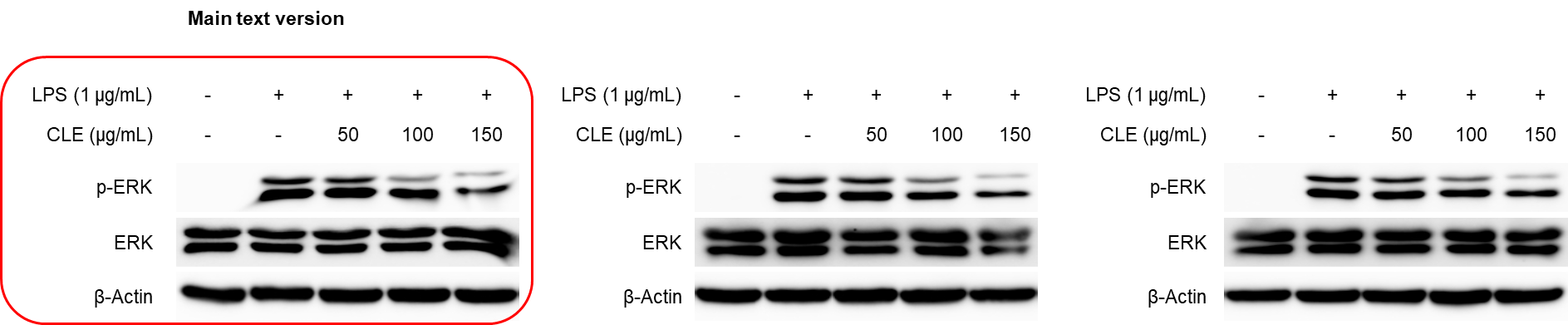


**Supplementary Figure 11.** Original Western blots for p-JNK (A), JNK (B), and corresponding β-actin proteins (C) in LPS-induced BV2 microglial cells. The images in which the membrane edges of the original blots are not clearly visible or appear to be cropped versions may be due to detection of protein in a short time because the proteins and primary antibodies interacted actively. The presented images are full-length, original, and unprocessed blots which were not cut prior to hybridization with antibodies during blotting.


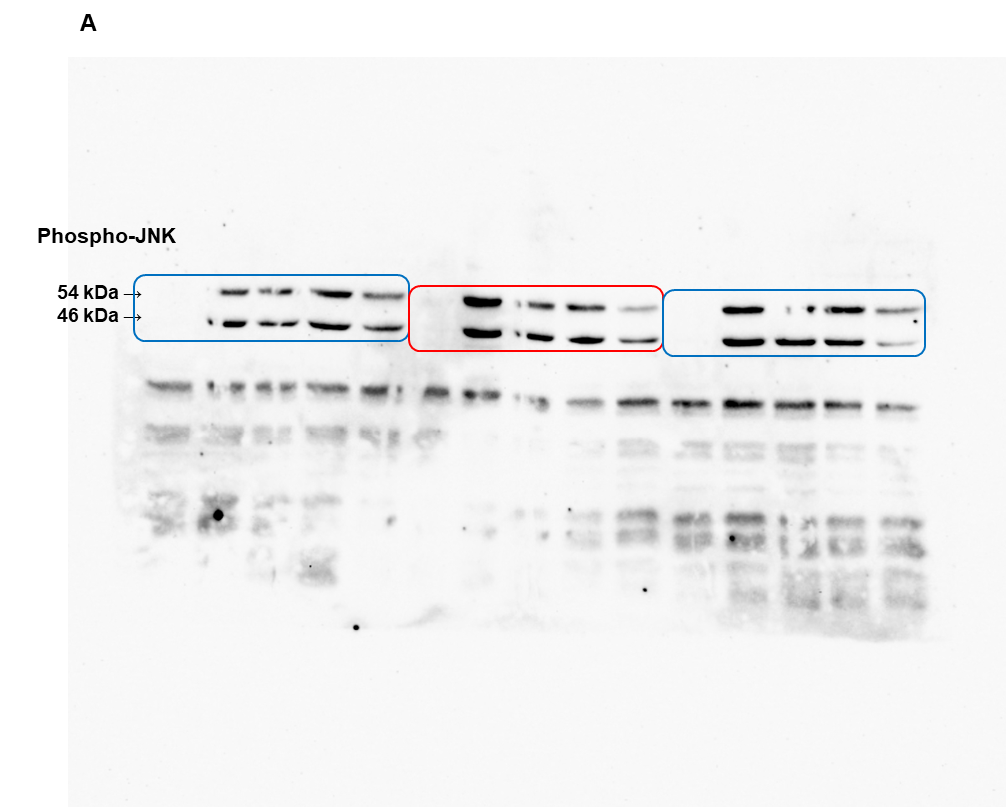


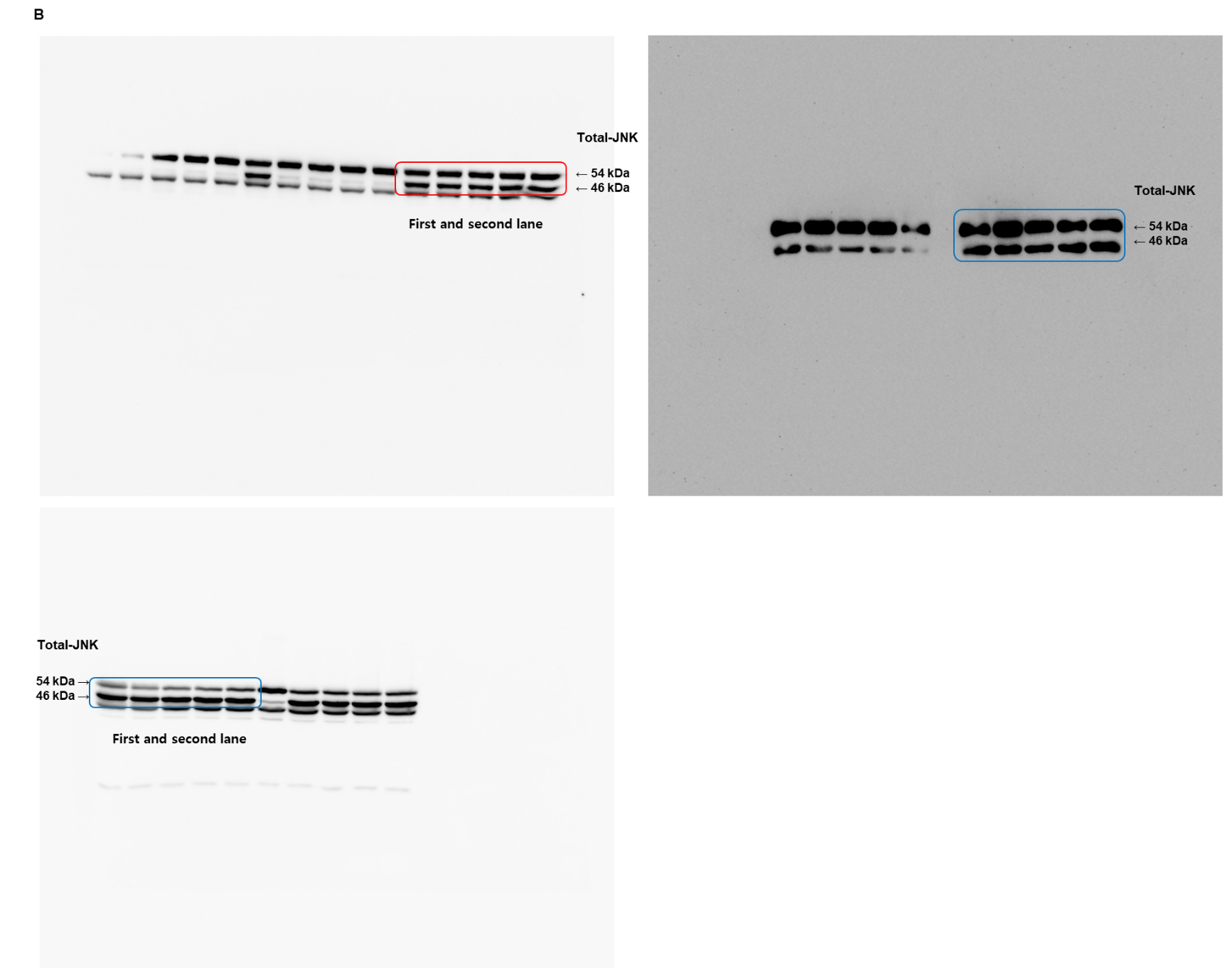


**
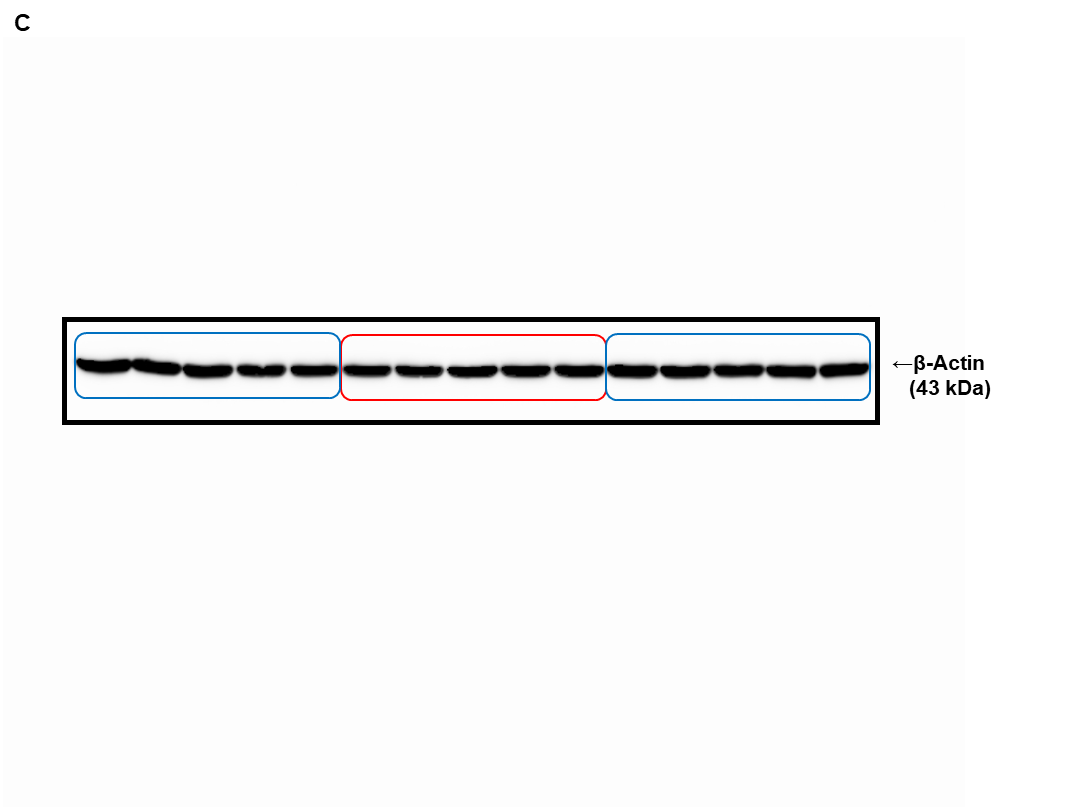
**

**Supplementary Figure 12.** Cropped version of p-JNK, total JNK, and their corresponding β-actin proteins in LPS-induced BV2 microglial cells pre-treated with CLE.

**
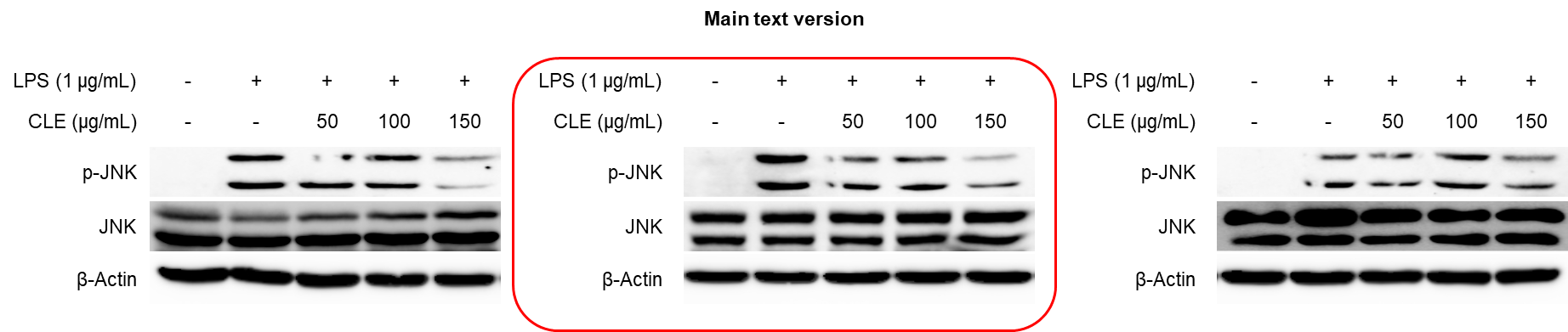
**

**Supplementary Figure 13.** Original Western blots for HO-1 and corresponding β-actin proteins in BV2 microglial cells.


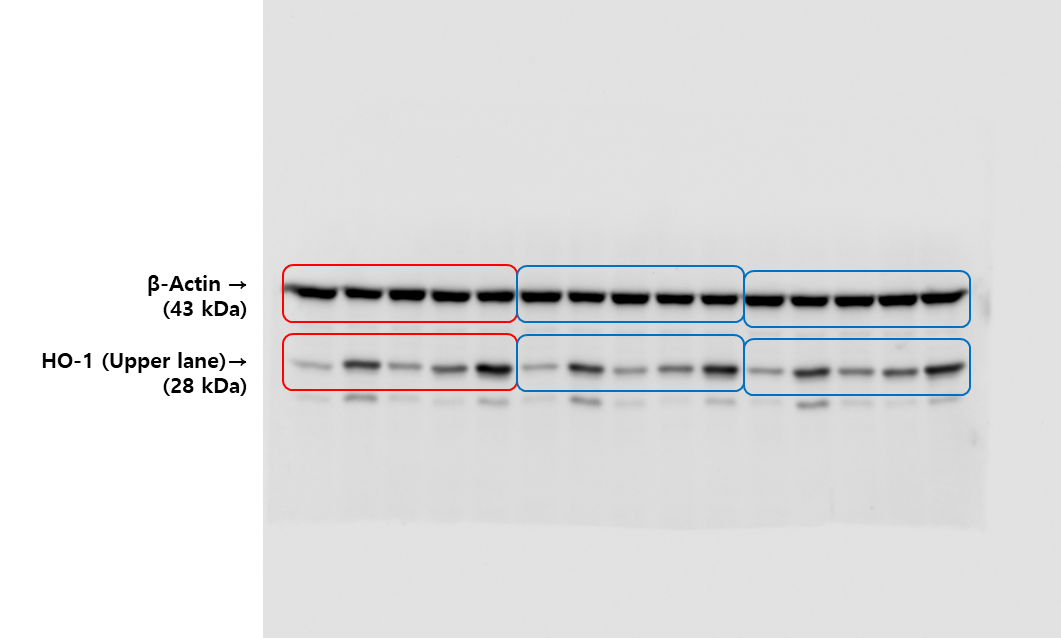


**Supplementary Figure 14.** Cropped version of HO-1 and corresponding β-actin proteins in BV2 microglial cells treated with CLE.

**
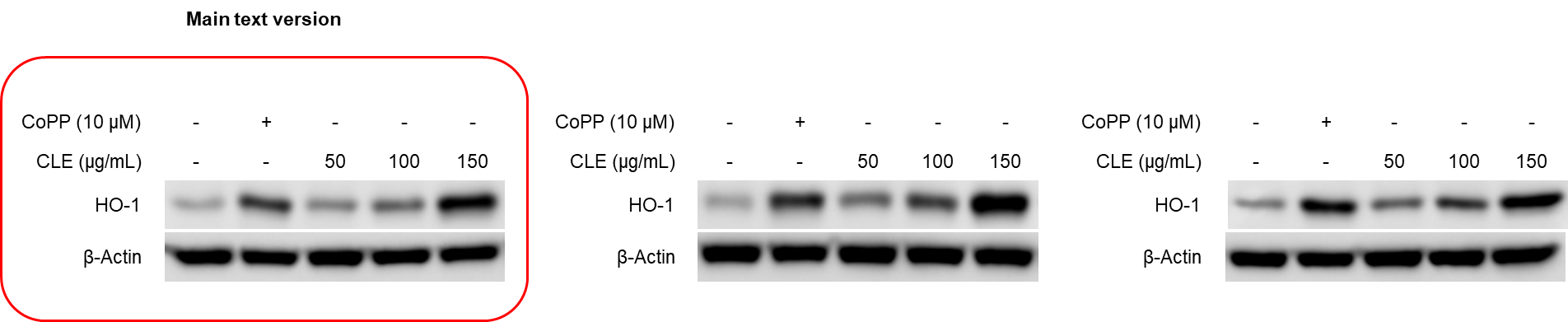
**

**Supplementary Figure 15.** Original Western blots for Nrf2 in cytoplasmic fraction (A) and corresponding β-actin proteins (B) BV2 microglial cells. The images in which the membrane edges of the original blots are not clearly visible or appear to be cropped versions may be due to detection of protein in a short time because the proteins and primary antibodies interacted actively. The presented images are full-length, original, and unprocessed blots which were not cut prior to hybridization with antibodies during blotting.


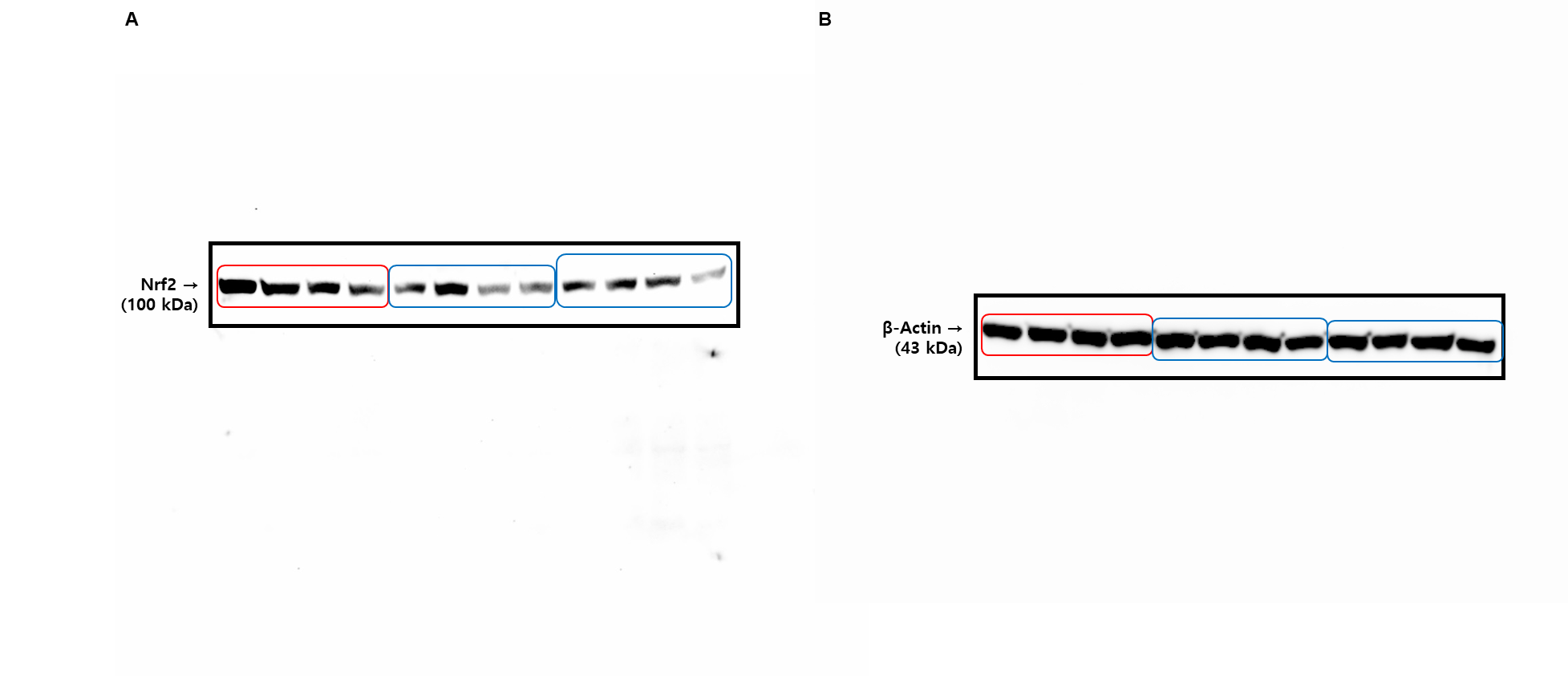


**Supplementary Figure 16.** Cropped version of cytosolic Nrf2 and corresponding β-actin proteins in BV2 microglial cells treated with CLE.

**
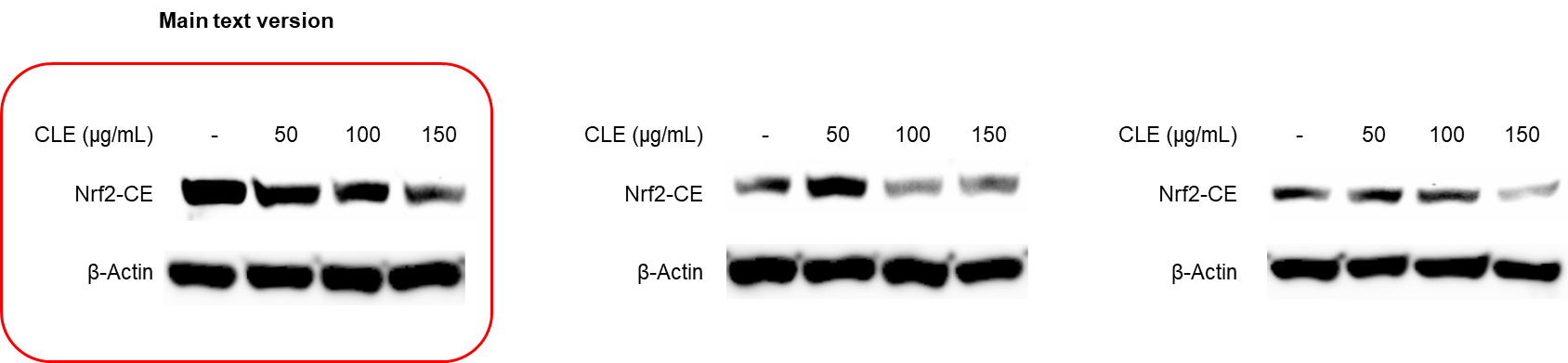
**

**Supplementary Figure 17.** Original Western blots for Nrf2 in nuclear fraction (A) and corresponding PCNA proteins (B) BV2 microglial cells. The images in which the membrane edges of the original blots are not clearly visible or appear to be cropped versions may be due to detection of protein in a short time because the proteins and primary antibodies interacted actively. The presented images are full-length, original, and unprocessed blots which were not cut prior to hybridization with antibodies during blotting.


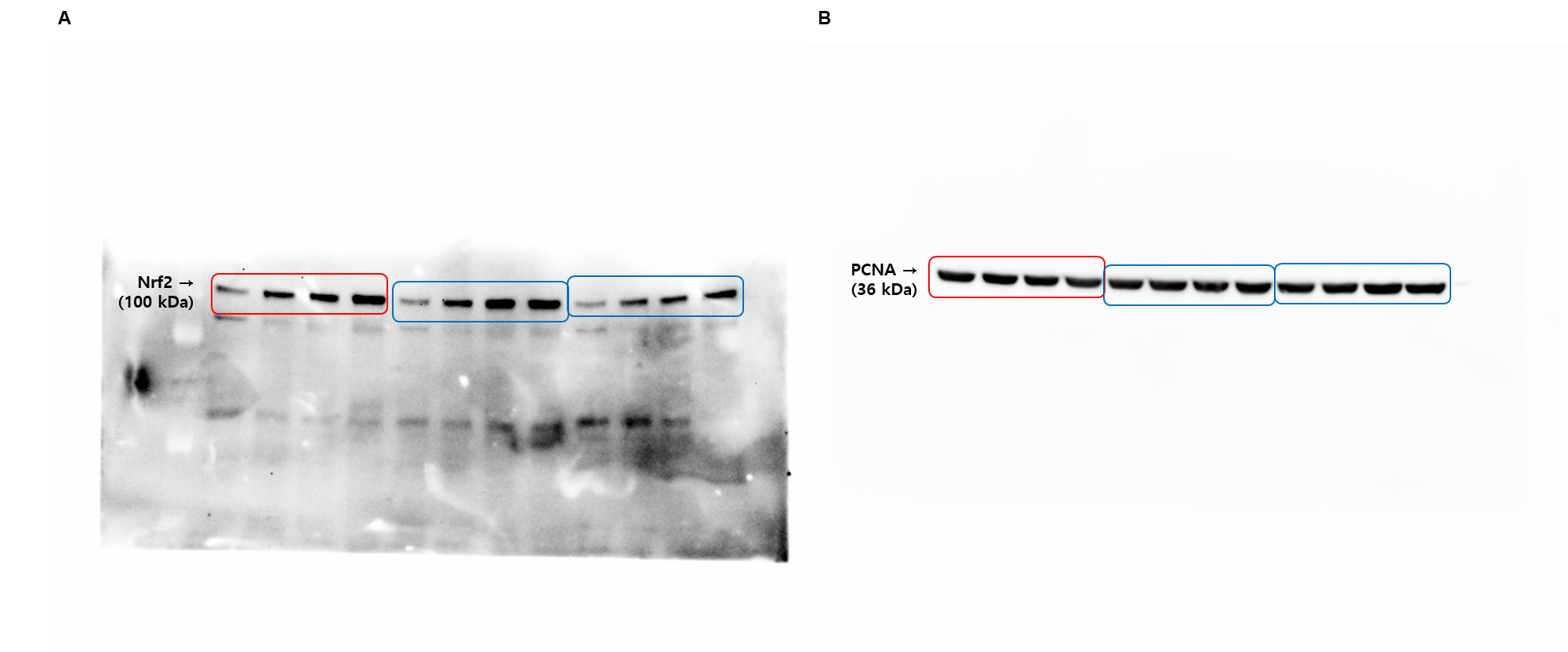


**Supplementary Figure 18.** Cropped version of nuclear Nrf2 and corresponding PCNA proteins in BV2 microglial cells treated with CLE.

**
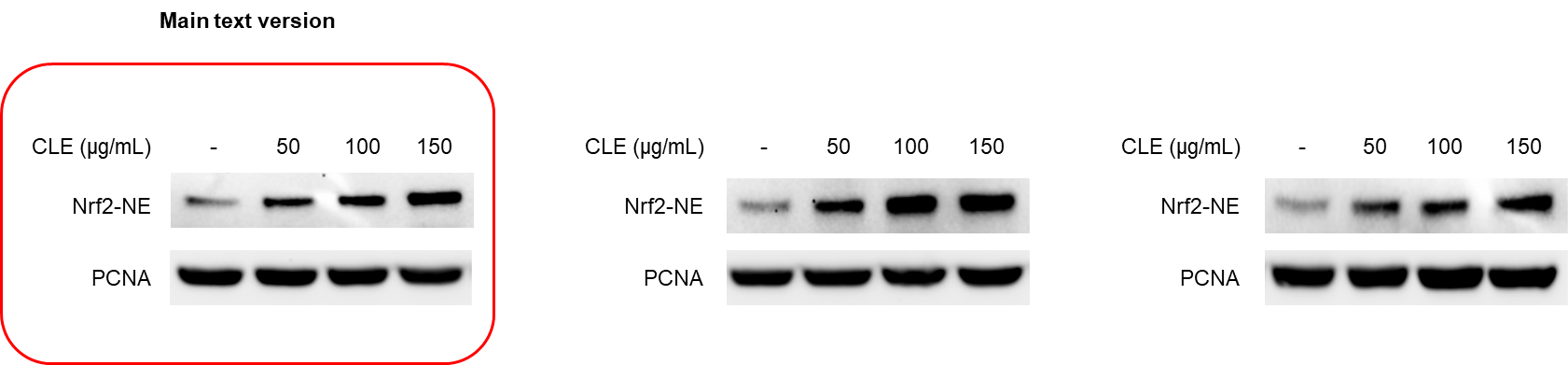
**

**Supplementary Figure 19.** Original Western blots for iNOS, COX-2 (A) and corresponding β-actin proteins (B) in LPS-induced BV2 microglial cells pre-treated with CLE SnPP. The images in which the membrane edges of the original blots are not clearly visible or appear to be cropped versions may be due to detection of protein in a short time because the proteins and primary antibodies interacted actively. The presented images are full-length, original, and unprocessed blots which were not cut prior to hybridization with antibodies during blotting.


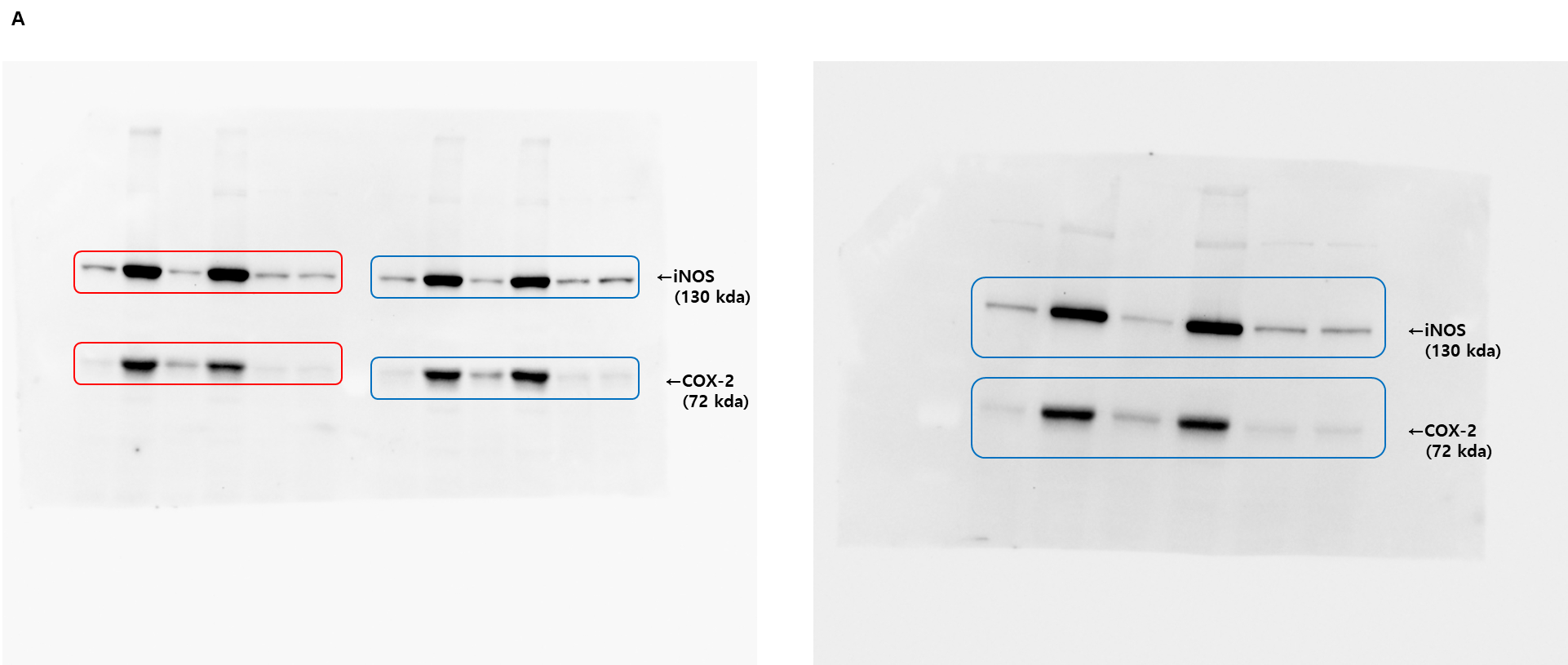


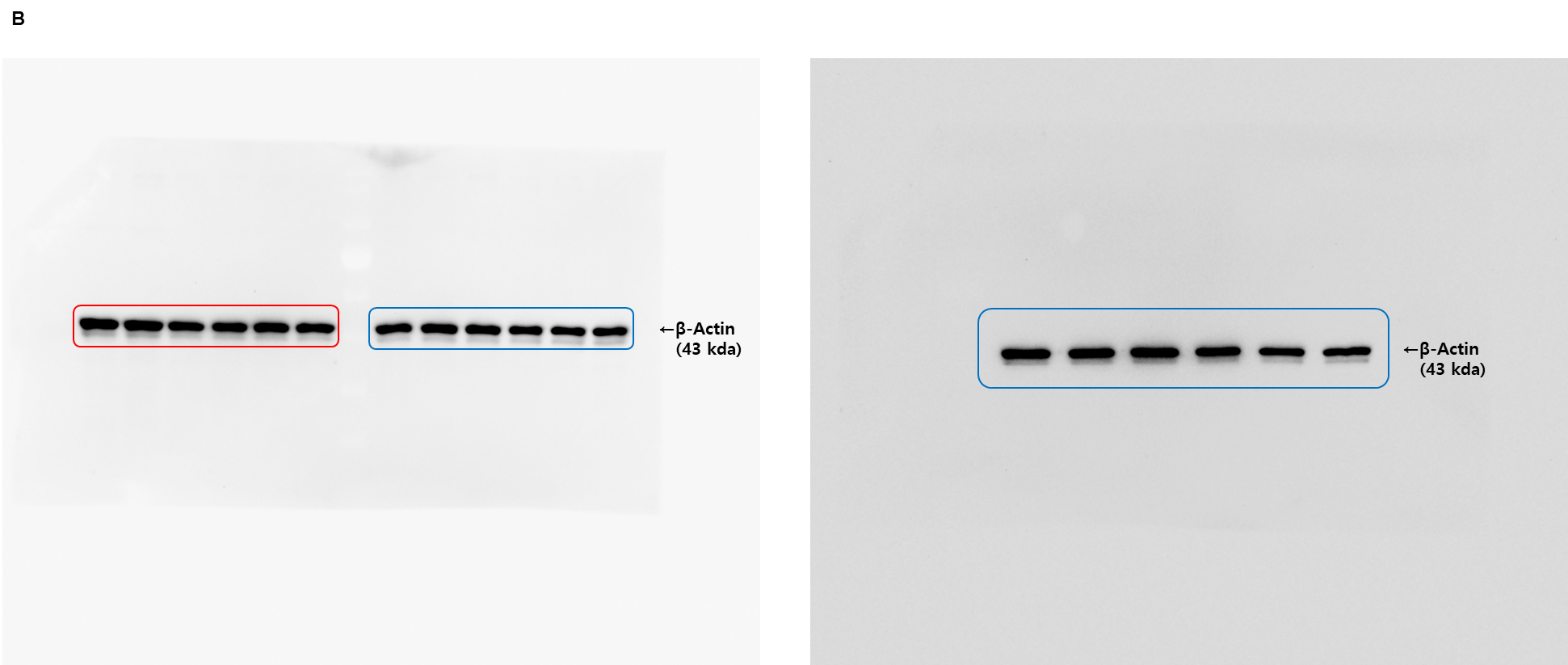


**Supplementary Figure 20.** Cropped version of iNOS, COX-2, and corresponding β-actin proteins in LPS-induced BV2 microglial cells pre-treated with CLE and SnPP.


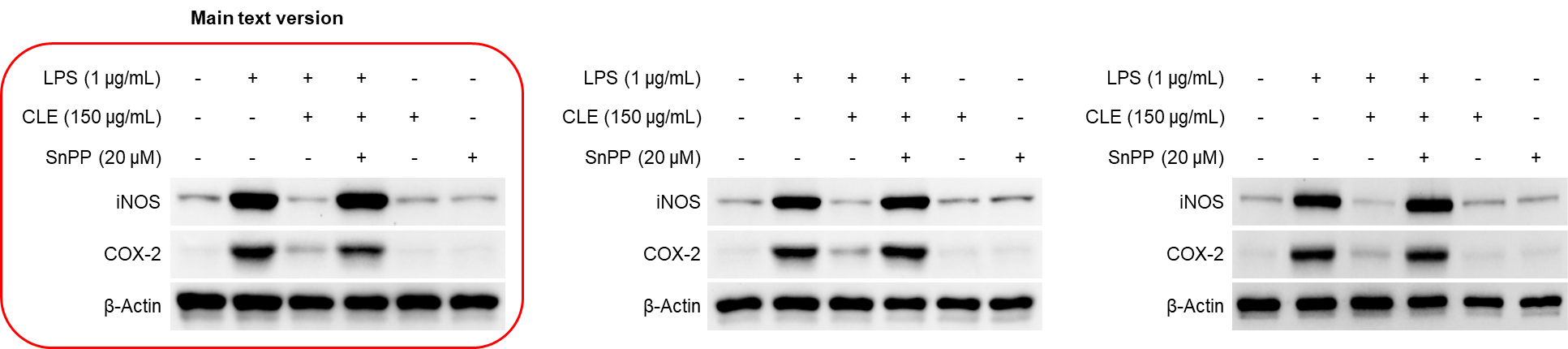

Supplement: Supplementary file 1 — Additional file 1. [file 12906_2022_3825_MOESM1_ESM.docx]
